# Modeling Short-Term Symptom Changes and Behavioral Subtypes of Depression and Anxiety in the General Population: Observational Study Using Smartphone Data

**DOI:** 10.2196/88083

**Published:** 2026-07-14

**Authors:** Sun Min Kim, Hyeon Gyu Park, Jae Wook Shin, Ji Hyu Park, Sung Woo Joo, Sungkyu Park, Dooyoung Jung, Sukhan Lee, Sang Won Lee, Hwang Kim, Young Tak Jo, Sungahn Ko, Ahyoung Choi, Jungwook Rhim, Jae Hyun Yoo, Jungsun Lee

**Affiliations:** 1 Department of Psychiatry, Asan Medical Center, 88 Olympic-ro 43-gil, Songpa-gu, Seoul, 05505, Republic of Korea, 82 2-3010-3422, 82 2-485-8381; 2 College of Medicine, University of Ulsan, Ulsan, Republic of Korea; 3 KDI School of Public Policy and Management, Sejong-si, Republic of Korea; 4 Department of Biomedical Engineering, Graduate School of Health Science and Technology, Ulsan National Institute of Science and Technology, Ulsan, Republic of Korea; 5 Department of Artificial Intelligence, Sungkyunkwan University, Suwon-si, Republic of Korea; 6 Department of Psychiatry, Kyungpook National University Chilgok Hospital, Daegu, Republic of Korea; 7 School of Medicine, Kyungpook National University, Daegu, Republic of Korea; 8 Department of Design, Ulsan National Institute of Science and Technology, Ulsan, Republic of Korea; 9 Department of Psychiatry, Kangdong Sacred Heart Hospital, Seoul, Republic of Korea; 10 College of Medicine, Hallym University, Chuncheon-si, Republic of Korea; 11 Computer Science and Engineering, Graduate of School of AI, Pohang University of Science and Technology, Pohang-si, Republic of Korea; 12 Department of AI and Software, Gachon University, Seongnam-si, Republic of Korea; 13 Department of Artificial Intelligence Convergence, Kangwon National University, Chuncheon, Republic of Korea; 14 Department of Psychiatry, The Catholic University of Korea, Seoul St Mary's Hospital, Seoul, Republic of Korea; 15 College of Medicine, Catholic University of Korea, Seoul, Republic of Korea

**Keywords:** digital health, psychiatry, cluster analysis, depression, anxiety, mobile phone

## Abstract

**Background:**

Smartphone-based digital phenotyping has emerged as a promising approach for monitoring mental health using passive behavioral data. Prior studies have linked smartphone-derived features to depression and anxiety severity; however, knowledge regarding whether short-term changes in symptoms can be captured using passive smartphone data in general population samples remains limited, as does the understanding of how such findings should be interpreted vis-à-vis behavioral patterns and demographic variability.

**Objective:**

This study aimed to model short-term changes in depression and anxiety severity using passive smartphone data, examine model performance across demographic subgroups, and identify behavioral patterns associated with symptom changes.

**Methods:**

We collected 2 weeks of smartphone usage data from 95 adults in the general population and assessed depressive and anxiety symptoms using the clinician-rated Hamilton Depression Rating Scale and Hamilton Anxiety Rating Scale, respectively. Behavioral features—including physical activity, app use, and screen usage metrics—were extracted and compressed using an autoencoder and principal component analysis. The resulting features—along with age, sex, and baseline Hamilton scores—were used to train random forest classifiers predicting symptom score changes (increase, decrease, or unchanged). Additionally, we examined whether model performance differed across demographic subgroups and whether models excluding baseline scores retained predictive performance, as baseline severity was expected to be a strong predictor. To add explanatory value beyond prediction, behavioral subtypes associated with symptom changes were identified by applying unsupervised clustering.

**Results:**

The model exhibited moderate performance in predicting changes in the Hamilton Depression Rating Scale (mean accuracy=0.70, mean area under the receiver operating characteristic curve=0.74) and Hamilton Anxiety Rating Scale (mean accuracy=0.65, mean area under the receiver operating characteristic curve=0.69) scores. Performance varied according to demographics, with reduced accuracy among younger adults and females, although these differences were not significant in permutation tests. Excluding baseline Hamilton scores diminished performance substantially, suggesting that baseline symptom severity accounted for a substantial proportion of the predictive performance. Clustering revealed 4 distinct behavioral subtypes according to smartphone usage patterns. A cluster characterized by structured, daytime-focused smartphone use and lower temporal entropy demonstrated greater improvement in depressive symptoms, whereas clusters with lower and irregular usage patterns exhibited minimal improvement or worsening.

**Conclusions:**

Passive smartphone-derived behavioral data demonstrated moderate ability to model short-term symptom changes in this predominantly nonclinical sample. However, a substantial proportion of the predictive performance was attributable to baseline symptom severity, underscoring that passive smartphone data may provide modest supplementary information rather than robust stand-alone predictive value. Nevertheless, clustering analyses indicated that passive data may still assist in identifying behaviorally distinct subtypes associated with different depressive symptom trajectories. These findings reflect a practical contribution to digital phenotyping research by elucidating both the potential and constraints of passive smartphone data for short-term symptom monitoring in small general population samples.

## Introduction

Smartphones, now ubiquitous in daily life, offer a promising platform for digital phenotyping—the moment-by-moment quantification of individual behavior using data from personal digital devices [1-3]. Recent studies have suggested that various features passively captured by smartphones, such as reduced mobility (eg, GPS location variability) and altered communication patterns (eg, call and texting frequency), are associated with depression and anxiety severity [4-8]. Depression and anxiety are common symptoms even among individuals in the general population who do not regularly visit mental health care services, and these symptoms are typically underrecognized and not routinely monitored [9,10]. Moreover, accumulating evidence has suggested that timely psychiatric intervention may benefit individuals with subthreshold symptoms that are not fully episodic [11,12]; although several prior studies have attempted to use digital data–based phenotyping for early detection and symptom tracking in the general population [13], a need remains for research that explicitly accounts for interindividual, demographic variability. Furthermore, beyond cross-sectional detection or prediction, studies examining whether within-individual changes in symptoms over time can be monitored using digital data have primarily been conducted in clinical populations such as patients with major depressive disorder [14-16]. Evidence is still limited on whether short-term dynamic changes in symptoms can be reliably modeled using digital data in general population samples.

Moreover, beyond prediction performance, understanding the behavioral patterns underlying symptom changes is critical [1], as psychiatric symptoms (eg, depression) manifest through complex and heterogeneous combinations of behavioral changes arising from diverse pathophysiological mechanisms [17]. However, existing predictive models in psychiatry generally provide limited interpretability regarding why specific classifications are made. Rather than capturing holistic behavioral patterns, numerous approaches rely on a small number of isolated features to explain symptom trajectories.

Accordingly, this study aimed to examine whether passively collected smartphone usage data are sensitive to short-term (2-week) within-individual changes in anxiety and depression scores in a predominantly nonclinical adult sample. Specifically, we sought to (1) develop a model classifying short-term increases, decreases, or stability in the Hamilton Depression Rating Scale (HAM-D) and Hamilton Anxiety Rating Scale (HAM-A) scores; (2) evaluate model performance across age and sex subgroups; and (3) identify data-driven behavioral patterns associated with differing symptom change trajectories. This study aims to examine digital phenotyping’s potential to capture behaviorally meaningful variability in everyday contexts, contributing to digital phenotyping research’s methodological and conceptual development.

## Methods

### Study Population and Data Collection

This study is a secondary analysis of prospectively collected data obtained from ongoing government-funded research projects that collected smartphone-based behavioral data and clinical symptom assessments. The data used for this analysis were obtained from adult volunteers (aged 19‐54 years) from the general population—recruited between June 1, 2024, and February 28, 2025, through on-site and online advertisements at multiple participating institutions in South Korea, including Asan Medical Center (AMC), Kyungpook National University Chilgok Hospital, Ulsan National Institute of Science and Technology, Kangdong Sacred Heart Hospital, and Sungkyunkwan University. Smartphone data were collected for research purposes using a custom-developed mobile app created by the research team [18]. Owing to compatibility requirements of the data collection app used herein, only those individuals using Android smartphones (Samsung Galaxy S8 or newer) were eligible for inclusion. Depression and anxiety symptoms were assessed using HAM-D and HAM-A, respectively, which are clinician-rated scales widely used to assess the severity of depressive and anxiety symptoms. Based on commonly used cutoff values, HAM-D scores of 0‐7, 8‐16, 17‐23, and ≥24 indicate no, mild, moderate, and severe depression, respectively [19], while HAM-A scores of 0‐7, 8‐14, 15‐23, and ≥24 indicate no or minimal, mild, moderate, and severe anxiety, respectively [20]. At study entry (baseline), the HAM-A and HAM-D were administered by board-certified psychiatrists or psychiatry residents under the supervision of psychiatrists, all of whom were registered investigators. Furthermore, HAM-A and HAM-D assessments were conducted in person for participants recruited from institutions with medically licensed investigators (Asan Medical Center, Ulsan National Institute of Science and Technology, Kangdong Sacred Heart Hospital, and Kyungpook National University Chilgok Hospital). Assessments were conducted through Zoom (Zoom Communications, Inc) by clinicians from AMC for participants recruited from Sungkyunkwan University. The same assessments were repeated after a short-term follow-up period of 2 weeks to measure changes in symptom severity. During follow-up, participants’ smartphone usage data were continuously collected using the aforementioned app, which passively recorded the following multimodal behavioral data: (1) physical activity and environmental context metrics (eg, distance traveled every 10 minutes and ambient light intensity by hour detected by smartphone sensors), (2) screen usage data by hour, and (3) app usage logs. Participants’ demographic information—including their age, sex, and prior psychiatric diagnosis—was collected at baseline. Considering a balance between temporal resolution and storage feasibility, most data were collected at hourly intervals, except for app usage logs and the moved distance. Multimedia Appendix 1 presents a detailed description of the raw data collected and their derived features. Changes in HAM scores and smartphone features were compared across age and sex groups using Kruskal-Wallis tests or Spearman correlation analyses. Participants whose follow-up clinician assessment was incomplete or whose smartphone data were insufficient (missing data for any day within the 14-day period or refusal to provide app usage information) were excluded from the analysis. We assessed potential selection bias attributable to participant exclusion by comparing baseline demographic and clinical characteristics (age, sex, and baseline and follow-up HAM-A and HAM-D scores) between included and excluded participants using Kruskal-Wallis tests and chi-square tests.

### Ethical Considerations

All study procedures were approved by the Institutional Review Board of the AMC (IRB no. 2022‐0638) and conducted in accordance with the Declaration of Helsinki. All participants provided written informed consent. Privacy and confidentiality were strictly safeguarded, while all smartphone-derived information was anonymized before analysis. Participants received transportation expense reimbursement only, with no additional compensation. A publicly accessible protocol for this secondary analysis was unavailable at the time of submission.

### Data Preprocessing and Feature Engineering

We extracted the following features for the analysis, which resulted in the following 20 variables: the daily sum of location displacement and ambient light levels, serving as indicators of physical activity and environmental context; daily app usage time based on category (classified according to the default categories provided by the Google Play Store), reflecting qualitative patterns of smartphone use; and screen time data (usage duration, the number of times the screen was turned on, and unlocks per hour), captured by dividing the day into 3 time intervals—specifically, day (9 AM to 5 PM), evening (5 PM to 1 AM), and night (1 AM to 9 AM)—representing temporal and quantitative usage patterns throughout the day. The day was divided into 3 fixed periods (9 AM to 5 PM, 5 PM to 1 AM, and 1 AM to 9 AM) to approximate structured daytime activity, evening leisure or social periods, and nighttime rest periods. The 9 AM boundary was selected to reflect common workday or school start times in the study population, thereby capturing routine-related temporal variation. All features were standardized (z-scored) at the individual level to capture within-individual variability rather than absolute values, considering that the predictive model aimed to estimate changes in HAM scores over the 2-week period. Multimedia Appendix 1 lists details of derived features used for subsequent analysis, and Multimedia Appendix 2 provides the anonymized derived feature data.

**Figure 1.**
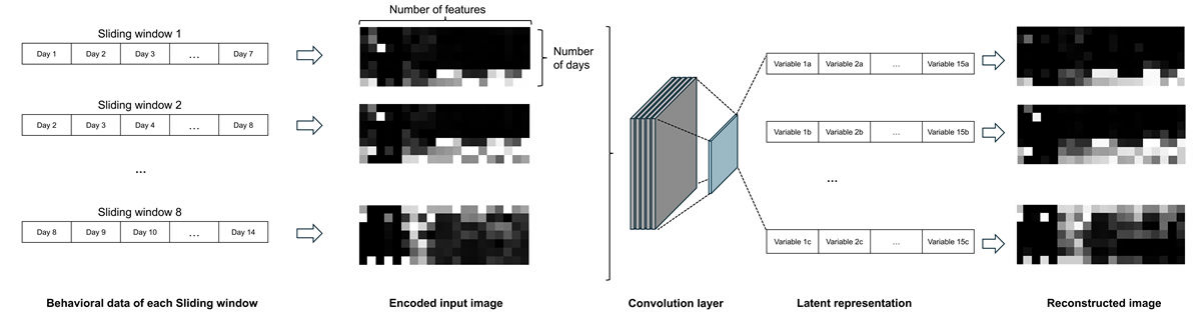
Overview of the data compression process using a convolutional autoencoder.

Studies using raw digital data collected over time commonly use various machine learning methods for feature compression. Following methodologies demonstrated in previous research [21], we used an autoencoder—a deep learning approach that encodes input data into a smaller set of latent variables—to convert time series data segments, structured as image-like inputs, into compact representations suitable for subsequent prediction tasks. To reflect temporal dynamics in the features while ensuring sufficient training of the autoencoder, we segmented each participant’s 14-day data into overlapping windows of 7 consecutive days using a sliding-window approach. For each participant, the 20 features from days 1‐7 were input as a single “image” into the autoencoder; furthermore, days 2‐8 were input as the subsequent image, and so on, up to days 8‐14. The window size of 7 days was selected to ensure that all days of the week were represented within each segment. Using this approach, 8 overlapping windows (14 days—window size, which is 7+1) were generated per participant. The autoencoder’s hyperparameters were selected from combinations minimizing the final reconstruction error after 20,000 training epochs with a batch size of 8. The final settings were as follows: a learning rate of 1e-4 with the Adam optimizer, a kernel size of 3 (vertical dimension), 64 convolutional filters, and 15 latent variables. Figure 1 depicts this data compression process using the convolutional autoencoder (CAE).

Each 7-day window comprised 140 variables (7 days×20 features) and was encoded into 15 latent variables—subsequently reduced to 4 dimensions using principal component analysis (PCA). We evaluated principal component counts from 2 to 10 and selected 4 as the final number based on a trade-off between explained variance and the risk of overfitting because of high dimensionality. This resulted in 32 variables (8 sliding windows × 4 compressed variables per window) for each participant, enabling us to represent complex and correlated behavioral metrics in a lower-dimensional feature space while preserving salient information [22]. The code used to process smartphone-derived metrics using the CAE is provided in Multimedia Appendix 3, and the processed values generated from this code are provided in Multimedia Appendix 4.

### Predictive Modeling

Considering our primary objective of developing a model to predict short-term changes, we designed the model to classify whether a participant’s HAM-A or HAM-D scores had increased, decreased, or remained unchanged compared with the baseline score obtained 2 weeks earlier. As predictors, we included 32 variables generated in the previous steps, alongside participants’ sex, age, and baseline HAM-A or HAM-D scores at week 0. The inclusion of baseline HAM scores is substantiated by previous studies identifying initial symptom severity as a key prognostic factor for subsequent changes in anxiety and depression severity [23]. We constructed a supervised machine learning model using a random forest classifier—selected for its robustness to noisy features, ability to capture nonlinear relationships, and prior evidence highlighting that ensemble tree-based methods typically perform effectively in mental health prediction tasks using passive data [24].

The random forest model comprised 500 decision trees trained on bootstrapped samples, with random feature selection at each split (mtry). The optimal mtry value was identified using grid search (tuneLength=10), whereas other hyperparameters (eg, maximum tree depth and minimum node size) were left at default settings to mitigate overfitting risk. Model performance was assessed using stratified 5-fold cross-validation, with 80% and 20% of the data used for training and testing in each fold, respectively, thereby ensuring a sufficiently large training set to learn model parameters while reserving an independent subset to obtain a stable estimate of predictive performance. The procedure was repeated 100 times to ensure stable performance estimates, with predictive accuracy, the area under the receiver operating characteristic curve (AUROC), and the *F*_1_-score calculated and averaged across all iterations. Separate random forest classifiers were trained for predicting changes in HAM-A and HAM-D scores. Model calibration was not assessed, as this study primarily aimed to classify the direction of short-term symptom change rather than to estimate individual event probabilities.

We conducted 2 sets of secondary analyses to further evaluate the robustness and interpretation of the primary random forest models. First, we examined whether model performance was influenced by the incomplete separation of training and test data during the CAE training stage. In the primary analysis, the CAE was trained using the full dataset before downstream predictive modeling. This approach was selected because the CAE was not used as a direct prediction model but rather as a representation learning tool intended to extract latent behavioral embeddings from smartphone-derived data. As we aimed to embed all participants within a common latent behavioral space, training the CAE on the full dataset was considered methodologically advantageous, particularly considering the modest sample size and the relatively small amount of time series data available for representation learning. Nevertheless, we additionally conducted the first secondary analysis by applying train-test separation before CAE training because incomplete train-test separation during this representation learning process may still raise concerns regarding information leakage. The CAE was trained only on the training data; subsequently, the test data were compressed using the trained CAE without updating model parameters. The remaining analytical steps were conducted using the same modeling procedure. This process was repeated 20 times with different random seeds, while the averaged performance metrics were compared with those of the primary model. We repeated the same train-test-separation-before-CAE procedure using penalized logistic regression instead of random forest classification to further assess whether the observed performance depended on the random forest classifier’s potential overfitting.

Second, we evaluated model performance after excluding baseline Hamilton scores from the predictor set because baseline HAM-A and HAM-D scores were included in the primary models and expected to be strong predictors of short-term symptom change. This comparison aimed to clarify the extent to which predictive performance reflected baseline symptom severity versus passive smartphone-derived behavioral information. The model excluding baseline scores was evaluated using the same train-test-separation framework: the CAE was trained only on the training data, test data were embedded using the trained CAE without parameter updates, and classification performance was averaged across 20 random seeds.

### Comparing Performance Across Demographic Subgroups

We address our second objective by examining model performance across demographic subgroups to identify potential disparities. Participants were stratified based on age group (younger adults ≤ median age versus older adults > median age) and sex (male vs female). Age was dichotomized using the sample median to ensure balanced group sizes and minimize potential bias arising from class imbalance, considering the relatively small sample size. The random forest model was evaluated separately for each subgroup using the aforementioned performance metrics (accuracy, AUROC, and *F*_1_-score—averaged across 100 iterations).

We evaluated statistical differences in model performance between subgroups using a permutation-based significance test (2000 permutations), wherein group labels were randomly shuffled while preserving group sizes. For each permutation, model training and evaluation were repeated 10 times to account for stochastic variation, while 2-sided *P* values were computed as the proportion of permuted differences exceeding the observed difference in absolute value.

### Clustering of Behavioral Patterns

We performed an exploratory clustering analysis using the autoencoder-derived latent representations of the smartphone-derived behavioral data (the data before PCA-based dimensionality reduction for the random forest model) to identify distinct behavioral patterns associated with mental health outcomes. Notably, the random forest model alone does not reveal which specific aspects of the smartphone usage patterns contributed to the predicted outcomes; hence, we adopted commonly used clustering methods using t-distributed stochastic neighbor embedding and k-means to cluster irregular raw digital data and identify meaningful behavioral patterns.

We applied t-distributed stochastic neighbor embedding to reduce the 15 autoencoder-derived latent features (representing a 7-day sliding window) into 2 dimensions, followed by k-means clustering. The optimal number of clusters (k) was determined by evaluating silhouette coefficients across k values from 3 to 8, running 100 iterations for each value. The k=4 configuration, which attained the highest silhouette score (0.4304) across all 600 trials and ranked second in terms of average silhouette score (0.4054) across repetitions, was, thus, selected as the optimal number of clusters.

As each participant had 8 sliding windows, and each window was assigned to 1 of 4 clusters, every participant had a sequence of 8 cluster labels (eg, 1-1-2-3-...). We identified the most representative cluster for each participant by applying the PageRank algorithm to these sequences; each participant’s dominant cluster was defined as the cluster with the highest PageRank value, indicating the cluster with the highest probability of being visited following multiple transitions. This approach is grounded in previous work [21] reporting that participants’ sequential cluster codes—representing latent space embeddings of sleep and daily activity patterns—can be conceptualized as a hidden Markov process wherein the subsequent state depends on the current state.

After assigning participants to 4 clusters, we compared symptom changes (ie, HAM-A and HAM-D scores) and demographic distributions across clusters using Kruskal-Wallis tests with post hoc analyses. We examined cluster-specific behavioral patterns by evaluating differences across clusters using the following summary measures derived from each participant’s 14-day data: (1) each feature’s total sum value over 14 days (to compare overall activity and app usage levels across clusters), (2) the mean proportion of temporal usage metrics allocated to each period across clusters (to characterize how usage was distributed across day, evening, and night), and (3) the Shannon entropy of each feature’s daily values (to quantify day-to-day regularity vs variability in behavior during 2 weeks).

### Statistical Analysis

The feature extraction and construction of latent variables using an autoencoder were performed in Python (version 3.6.6; Python Software Foundation), following the methodology and open-source code provided by Park et al [21]. All subsequent analyses—including random forest modeling, subgroup comparisons, and clustering—were conducted in R (version 4.2; R Core Team). Statistical significance was based on an α value of <.05. Considering the presence of outliers in several smartphone-derived features and Hamilton assessment scores, we used nonparametric tests throughout, including the Kruskal-Wallis rank sum test for group comparisons and Spearman correlation for continuous associations. The Kruskal-Wallis rank sum test was followed by pairwise Dunn test, with *P* values adjusted using the Bonferroni method.

## Results

### Participant Characteristics

The analysis used a subset of data collected during an ongoing study’s early phase. From June 1, 2024, to February 28, 2025, among the 158 participants enrolled during this period, 63 were excluded owing to missing data, resulting in a final analytic sample of 95 participants. Among those excluded, 52 did not provide consent for app activity monitoring, which prevented the collection of smartphone app usage data, as the user authorization required for app usage tracking was not actively monitored in the study application’s initial version. Notably, 9 participants experienced intermittent data loss because of app-related network errors that resulted in missing smartphone usage records for several days, while 2 had missing demographic information (age or sex). Table S1 in Multimedia Appendix 1 presents comparisons of baseline demographic and clinical characteristics of included and excluded participants. Among the 95 participants, 94 had no prior psychiatric diagnosis, while 1 had been diagnosed with major depressive disorder. Participants’ ages ranged from 19 to 54 years, with a mean of 30.36 (SD 9.50) years; the sample comprised 51 (54%) males and 44 (46%) females. Table S2 in Multimedia Appendix 1 presents additional participant demographics.

At baseline, the sample’s mean HAM-D and HAM-A scores were 2.48 (SD 3.63) and 2.41 (SD 3.82), respectively. At short-term follow-up (after 2 weeks), the mean HAM-A and HAM-D scores were 1.92 (SD 3.65) and 1.99 (SD 3.78). The change in HAM scores—the week 2 score minus the baseline score—ranged from −10 to +7 for HAM-A (mean −0.57, SD 2.32) and from −6 to +7 for HAM-D (mean −0.42, SD 2.06). HAM-A scores increased, decreased, and remained unchanged among 14 (14.7%), 34 (35.8%), and 47 (49.5%) participants, respectively. HAM-D scores increased, decreased, and remained unchanged among 16 (16.8%), 34 (35.8%), and 45 (47.4%) participants, respectively. Among participants with increased scores, the average change was +2.71 (SD 2.02) and +2.44 (SD 1.93) points for HAM-A and HAM-D, respectively. Among those with decreased scores, the average change was −2.71 (SD 1.99) and −2.32 (SD 1.55) points for HAM-A and HAM-D, respectively. Figure S1 in Multimedia Appendix 1 presents the distributions of changes in HAM-A and HAM-D scores across individuals.

When score changes were compared by sex, males exhibited a greater reduction in HAM-A than females (Kruskal-Wallis rank sum test, *χ*²_1_=6.6; *P*=.01), although the mean difference was small (approximately 1 point), limiting its clinical relevance. All other comparisons—including HAM-D change based on sex, age correlations, and age group differences—were not significant. Tables S3a and S3b in Multimedia Appendix 1 present the detailed results.

Furthermore, we examined whether smartphone usage metrics exhibited significant age and sex differences over the 2-week observation period. Comparing total smartphone usage time over the 14-day period—the sum of screen use duration across day, evening, and night periods—revealed that males demonstrated significantly higher usage than females. Comparing age groups revealed that the younger group exhibited a higher mean total usage; however, the rank-based comparison using the Kruskal-Wallis rank sum test indicated greater overall usage in the older group, suggesting that some high-usage individuals in the younger group may have skewed the mean. A similar pattern emerged in the comparison of temporal usage metrics: whereas these indicators’ mean values were generally higher in the younger group, the rank-based comparisons consistently demonstrated greater overall usage distributions in the older group (Table S4 and Figures S2 and S3 in Multimedia Appendix 1).

### Predictive Model Performance

The random forest models demonstrated moderate predictive performance for short-term changes in anxiety and depression. Using repeated 5-fold cross-validation with 100 iterations, the model’s performance metrics were averaged across all runs. The model yielded a mean accuracy of 0.65 (SD 0.02) for HAM-A (mean AUROC 0.69, SD 0.02; mean *F*_1_-score 0.60, SD 0.10) and 0.70 (SD 0.02) for HAM-D (mean AUROC 0.74, SD 0.02; mean *F*_1_-score 0.61, SD 0.09). Considering the 3-class prediction task, the observed performance exceeded equal probability chance expectations; however, accuracy should also be interpreted in relation to the majority-class baseline because nearly half of the participants remained unchanged.

In the first secondary analysis, the train-test split was applied before CAE training to evaluate whether incomplete separation during the representation learning stage influenced model performance. The performance metrics were largely comparable with those of the main analysis (mean values across 20 splits were 0.68 [SD 0.09] for accuracy, 0.72 [SD 0.12] for AUROC, and 0.71 [SD 0.10] for *F*_1_-score in the HAM-A model, and 0.67 [SD 0.11] for accuracy, 0.72 [SD 0.10] for AUROC, and 0.72 [SD 0.09] for *F*_1_-score in the HAM-D model). For most performance metrics, the estimates from the main analysis fell within 1 SD of the sensitivity analysis results, except for the *F*_1_-score, which exhibited slightly higher mean values in the sensitivity analysis. This suggests that data leakage during the CAE training stage did not substantially inflate model performance in the main analysis, although moderate variability in performance across different train-test splits was observed, indicating that model performance was somewhat influenced by the training and test data’s specific composition. Table S5 in Multimedia Appendix 1 presents the detailed results. We assessed whether this variability was attributable to overfitting of the random forest model by repeating the analysis using a penalized logistic regression model; comparable levels of variability across splits were observed, with similar standard deviations (Table S6 in Multimedia Appendix 1).

In the second secondary analysis, baseline Hamilton scores were excluded from the predictor set while retaining the same train-test-separation-before-CAE framework. Model performance diminished substantially. For HAM-A, the mean accuracy, AUROC, and *F*_1_-score were 0.53 (SD 0.08), 0.59 (SD 0.11), and 0.53 (SD 0.10), respectively; for HAM-D, the corresponding values were 0.47 (SD 0.10), 0.57 (SD 0.09), and 0.49 (SD 0.10), respectively. Table 1 presents comparisons of performance metrics between models with and with no baseline Hamilton scores, while Table S7 in Multimedia Appendix 1 provides detailed results of 20 iterations of the model without baseline scores.

**Table 1.** Predictive performance of models with and with no baseline Hamilton scores.

Model specification	Accuracy	AUROC^a^	*F*_1_-score
HAM-A^b^ primary model (including baseline HAM-A), mean (SD)	0.65 (0.02)	0.69 (0.02)	0.60 (0.10)
HAM-A model excluding baseline score, mean (SD)	0.53 (0.08)	0.59 (0.11)	0.53 (0.10)
HAM-D^c^ primary model (including baseline HAM-D), mean (SD)	0.70 (0.02)	0.74 (0.02)	0.61 (0.09)
HAM-D model excluding baseline score, mean (SD)	0.47 (0.10)	0.57 (0.09)	0.49 (0.10)

a AUROC: area under the receiver operating characteristic curve.

b HAM-A: Hamilton Anxiety Rating Scale.

c HAM-D: Hamilton Depression Rating Scale.

### Comparing Performance Across Demographic Subgroups

We observed some variation in model performance across demographic subgroups (Tables2  3). Notably, the predictive accuracy was lower among younger participants (aged 27 years and younger, which is the median age) in both the HAM-A and HAM-D prediction models. The HAM-A classifier achieved a mean accuracy of 0.50 (mean AUROC 0.66, mean *F*_1_-score 0.49) among younger participants and 0.70 (mean AUROC 0.64, mean *F*_1_-score 0.72) among older participants. Likewise, the HAM-D model’s performance was generally stronger among older participants than among younger participants. The subgroups were similar in size (male: n=51; female: n=44; age groups defined by the median split: n=48 and n=47), rendering it unlikely that group size imbalance substantially influenced the model performance.

**Table 2.** Model performance and permutation test results based on age group.

Metric	Younger (≤27 years) group, mean (SD)	Older (>27 years) group, mean (SD)	Observed difference	*P* value
HAM-A^a^ model				
Accuracy	0.50 (0.03)	0.70 (0.04)	−0.2	.12
AUROC^b^	0.66 (0.04)	0.64 (0.03)	0.02	.85
*F*_1_-score	0.49 (0.07)	0.72 (0.05)	−0.23	.21
HAM-D^c^ model				
Accuracy	0.64 (0.04)	0.71 (0.02)	−0.06	.61
AUROC	0.74 (0.02)	0.64 (0.04)	0.1	.4
*F*_1_-score	0.58 (0.05)	0.70 (0.04)	−0.13	.47

a HAM-A: Hamilton Anxiety Rating Scale.

b AUROC: area under the receiver operating characteristic curve.

c HAM-D: Hamilton Depression Rating Scale.

**Table 3.** Model performance and permutation test results based on sex group.

Metric	Male group, mean (SD)		Female group, mean (SD)		Observed difference	*P* value	
HAM-A^a^ model							
Accuracy	0.67 (0.03)		0.53 (0.04)		0.13	.32	
AUROC^b^	0.66 (0.04)		0.66 (0.05)		−0.01	.95	
*F*_1_-score	0.69 (0.04)		0.45 (0.06)		0.23	.20	
HAM-D^c^ model							
Accuracy	0.74 (0.04)		0.71 (0.03)		0.02	.88	
AUROC	0.73 (0.03)		0.68 (0.05)		0.05	.68	
*F*_1_-score	0.64 (0.06)		0.70 (0.08)		−0.07	.73	

a HAM-A: Hamilton Anxiety Rating Scale.

b AUROC: area under the receiver operating characteristic curve.

c HAM-D: Hamilton Depression Rating Scale.

Concerning sex differences, discrepancies were more pronounced in the HAM-A model, whereas the HAM-D model demonstrated comparable results between males and females. The HAM-A prediction model yielded a mean accuracy of 0.67 (mean AUROC 0.66, mean *F*_1_-score 0.69) for males and 0.53 (mean AUROC 0.66, mean *F*_1_-score 0.45) for females. The HAM-D model exhibited a mean accuracy of 0.74 (mean AUROC 0.73, mean *F*_1_-score 0.64) for males and 0.71 (mean AUROC 0.68, mean *F*_1_-score 0.70) for females.

These sex-based subgroup performance metrics were obtained by averaging performance across 100 iterations of random forest training. The predictor set remained consistent across models, except that sex and age were excluded in the sex- and age group–based comparisons, respectively. We assessed these observed differences’ significance by conducting permutation tests using accuracy, AUROC, and *F*_1_-score as dependent variables. However, the permutation tests did not identify significant differences between the subgroups, suggesting that the aforementioned observed differences reflect random variation rather than systematic effects.

### Behavioral Pattern Clustering Results

The clustering analysis revealed 4 clusters characterized by distinct patterns of behavioral data. Cluster 1 was characterized by relatively low overall smartphone usage and activity levels, accompanied by irregular temporal usage patterns. Cluster 2 demonstrated a broadly similar level of overall activity and smartphone usage to cluster 1 but exhibited relatively greater evening smartphone usage and differences across several app categories. Cluster 3 demonstrated relatively high overall smartphone usage and activity levels with a relatively balanced temporal distribution across the day. By contrast, cluster 4 exhibited high levels of smartphone usage and activity combined with a more structured temporal pattern, characterized by concentrated daytime usage and relatively reduced evening usage. Table 4 summarizes each cluster’s defining behavioral and clinical characteristics.

**Table 4.** Summary of behavioral and clinical characteristics across clusters.

Cluster	Activity and overall smartphone use	Temporal entropy (regularity)	Temporal usage pattern	Mean HAM-D^a^ change (SD)	Interpretation
1	Low	High (irregular)	Throughout the day	0.12 (2.52)	Low activity; irregular smartphone usage with a higher late-hour proportion
2	Low	High (irregular)	Throughout the day; higher evening proportion	−0.26 (1.31)	Similar to C1^b^, but with differences in major app categories
3	High	Moderate	Balanced	−0.40 (1.85)	Balanced behavior; high overall smartphone usage
4	High	Low (regular)	Daytime-focused	−1.86 (2.44)	Highly active, structured daytime routine

a HAM-D: Hamilton Depression Rating Scale.

b C1: cluster 1.

**Figure 2.**
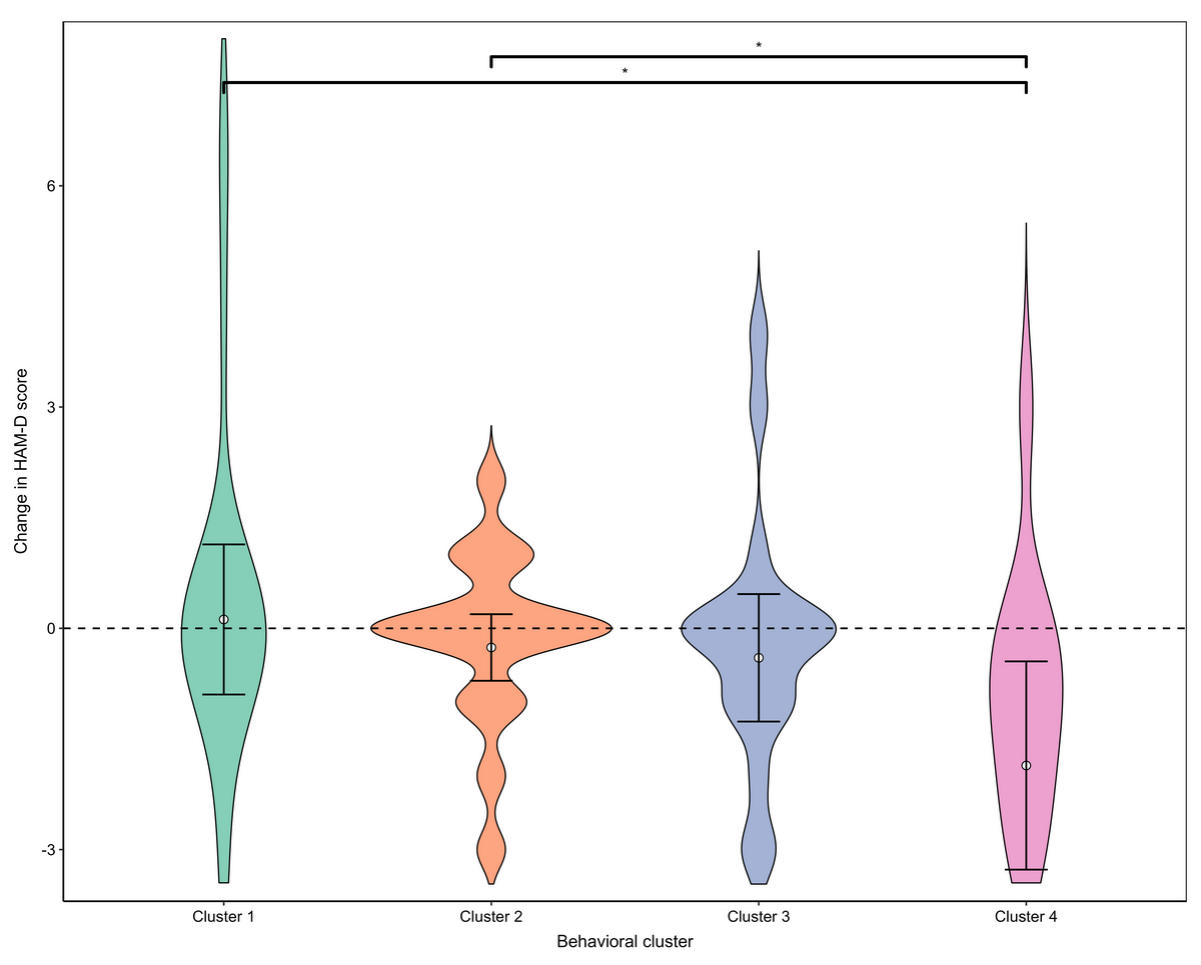
Distributions of HAM-D score changes across behavioral clusters. Violin plots depict the distribution of individual changes in HAM-D scores across behavioral clusters. White circles represent cluster means, while vertical lines indicate 95% CIs. Between-cluster differences were assessed using the Kruskal-Wallis test. Asterisks indicate Bonferroni-adjusted pairwise Dunn test results: *P<.05, **P<.01, and ***P<.001. Only significance levels corresponding to the observed pairwise comparisons are displayed. HAM-D: Hamilton Depression Rating Scale.

We examined whether changes in HAM scores exhibited significant differences across the identified clusters. No notable differences were observed across clusters for HAM-A (Kruskal-Wallis test, *χ*^2^_3_=2.7; *P*=.43), whereas differences between clusters were more pronounced for HAM-D (Kruskal-Wallis test, *χ*^2^_3_=9.6; *P*=.02); on average, cluster 1 (n=26) exhibited a slight increase in HAM-D scores (mean change 0.12, SD2.52), whereas clusters 2 (n=35; mean change −0.26, SD 1.31) and 3 (n=20; mean change −0.40, SD 1.85) demonstrated modest decreases. Cluster 4 (n=14) exhibited a substantial reduction (mean change −1.86, SD 2.44). Dunn post hoc test revealed that changes in HAM-D scores differed significantly between clusters 1 and 4 (*z*=−2.80; adjusted *P*=.02) and between clusters 2 and 4 (*z*=−2.87; adjusted *P*=.02); however, no other pairwise comparisons between clusters exhibited significant differences in changes in HAM-D scores (Figure 2).

For app usage-related variables, cluster 4 demonstrated higher values than the other groups across multiple domains, particularly in categories characterized by overall high usage and activity-related functions, such as productivity (mean 114.0, SD 138.6 hours over 14 days) and social apps (mean 194.5, SD 283.6 hours). Cluster 1 generally exhibited the lowest usage across most categories (eg, mean usage of 50.7 [SD 127.0] hours for productivity apps, 85.8 [SD 142.2] hours for social apps); cluster 2 exhibited a similar pattern to cluster 1 (eg, mean usage of 29.9 [SD 30.9] hours for productivity apps, 66.2 [SD 116.7] hours for social apps) but was distinguished by relatively higher usage in categories such as image apps (mean 0.4 [SD 1.3] hours), demonstrating low usage in the other clusters (mean 0.1 [SD 0.4] hours for cluster 1, mean <0.1 [SD<0.1] hours for cluster 3, and mean 0.1 [SD 0.2] hours for cluster 4). Cluster 3 exhibited moderate usage (second or third among clusters) in most domains but the highest usage in the video category (mean 133.2, SD 404.8 hours), with a significant difference compared with clusters 1 (mean 34.7 [SD 82.7] hours, Dunn post hoc test *z*=−8.30; adjusted *P*<.001) and 2 (mean 32.3 [SD 59.0] hours, *z*=−3.31; adjusted *P*=.001).

**Figure 3.**
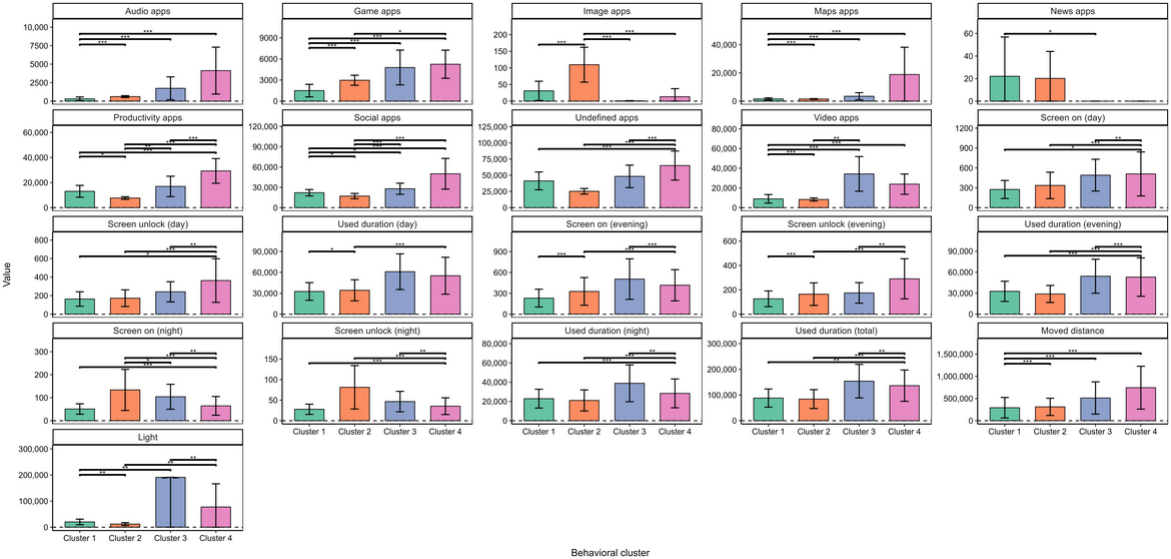
Comparisons of mean behavioral metrics across clusters. Bars depict cluster means with 95% CIs. Bar colors indicate cluster 1 (green), cluster 2 (orange), cluster 3 (blue), and cluster 4 (pink). Asterisks indicate Bonferroni-adjusted pairwise Dunn test results: *P<.05, **P<.01, and ***P<.001. Only significance levels corresponding to the observed pairwise comparisons are displayed. In the Light category, the CI for cluster 3 was excessively large; thus, the upper portion was truncated.

Among the activity-related indicators, the mean of cumulative daily moving distance increased in the following order: cluster 1 (mean 292, SD 1189 km) < cluster 2 (mean 312, SD 1382 km) < cluster 3 (mean 512, SD 1599 km) < cluster 4 (mean 744, SD 1403 km). Cluster 1, exhibiting the lowest value, demonstrated a significant difference compared with all 3 other clusters (Dunn post hoc test *z*=−7.58, adjusted *P*<.001; *z*=−6.84, adjusted *P*<.001; and *z*=−5.32, adjusted *P*<.001 for pairwise comparison with clusters 2, 3, and 4, respectively). For the cumulative sum of ambient light levels during 14 days, the mean value was highest in cluster 3 (mean 17.8, SD 19.3; log-transformed lux); however, owing to the presence of several outliers, Dunn test based on rank did not reveal a significant difference compared with cluster 2 (mean 12.0 [SD 13.4], *z*=−0.66; adjusted *P*=.51), which exhibited the lowest mean (Figure 3).

**Figure 4.**
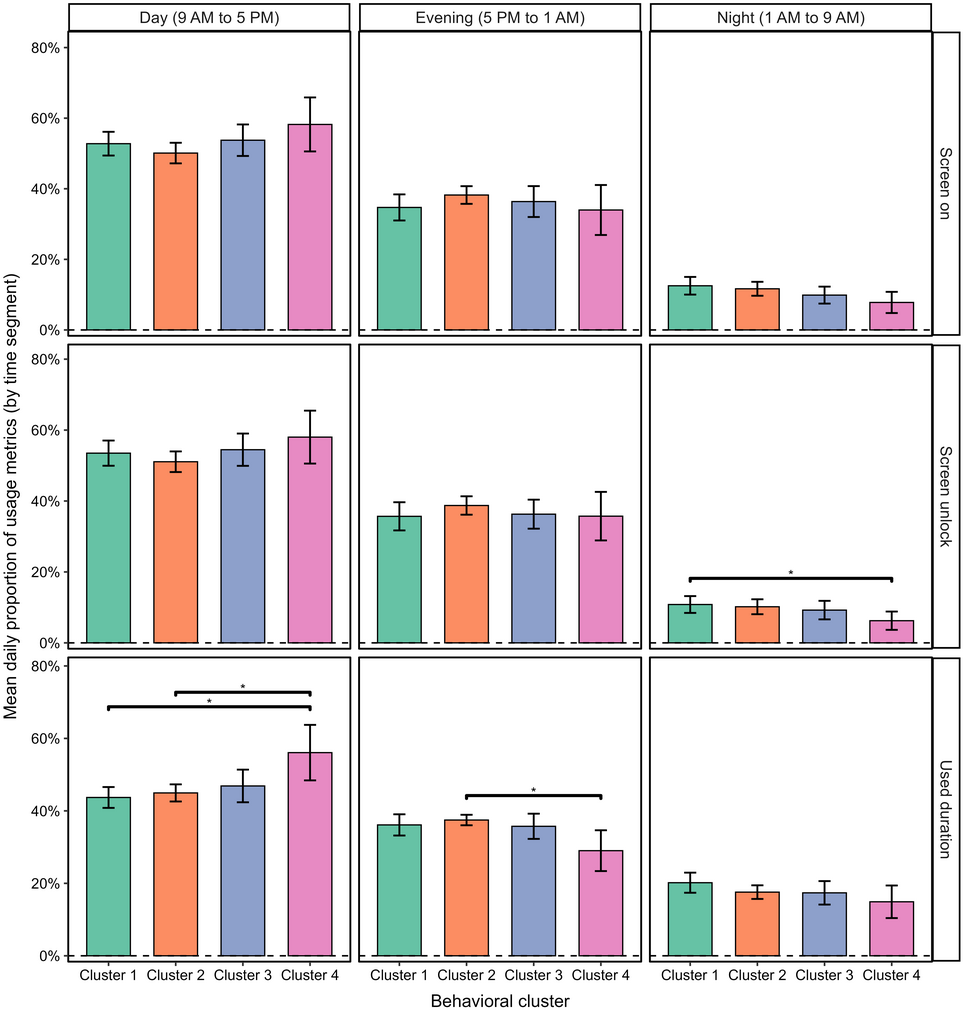
Temporal distribution of usage metrics based on behavioral clusters. Bars represent mean daily proportions of metrics by period (day, evening, and night) across clusters, with error bars indicating 95% CIs. Bar colors indicate cluster 1 (green), cluster 2 (orange), cluster 3 (blue), and cluster 4 (pink). Asterisks indicate Bonferroni-adjusted pairwise Dunn test results: *P<.05, **P<.01, and ***P<.001. Only significance levels corresponding to the observed pairwise comparisons are displayed.

For time-specific screen use metrics, which were segmented into day (9 AM to 5 PM), evening (5 PM to 1 AM), and night (1 AM to 9 AM), we differentiated temporal usage distribution patterns by comparing not only raw values (Figure 3) but also the average proportion of each screen usage metric (screen-on count, unlock count, and usage duration) during each period—specifically, the percentage of total daily usage allocated to each time segment by participants within each cluster—across clusters (Figure 4). When examining the proportion of usage duration by period, cluster 4 exhibited the highest proportion during the daytime (mean 0.56, SD 0.13) and the lowest proportion during the evening (mean 0.29, SD 0.10) and night (mean 0.15, SD 0.08), compared with all other clusters. Cluster 4 exhibited a significantly lower proportion of usage time during the evening than cluster 2 (Dunn post hoc test *z*=2.94; adjusted *P*=.02) and a significantly higher proportion during the day than clusters 1 (*z*=−3.07; adjusted *P*=.001) and 2 (*z*=−2.65; adjusted *P*=.05). By contrast, clusters 1 and 2 exhibited a relatively evenly distributed usage pattern throughout the day and higher nighttime usage proportions, although these differences were not significant. For the other 2 metrics (screen-on count and unlock count), generally similar trends were observed; however, the only marginally significant difference was observed in the nighttime unlock proportion, which differed between clusters 1 and 4 (*z*=2.64; adjusted *P*=.05).

**Figure 5.**
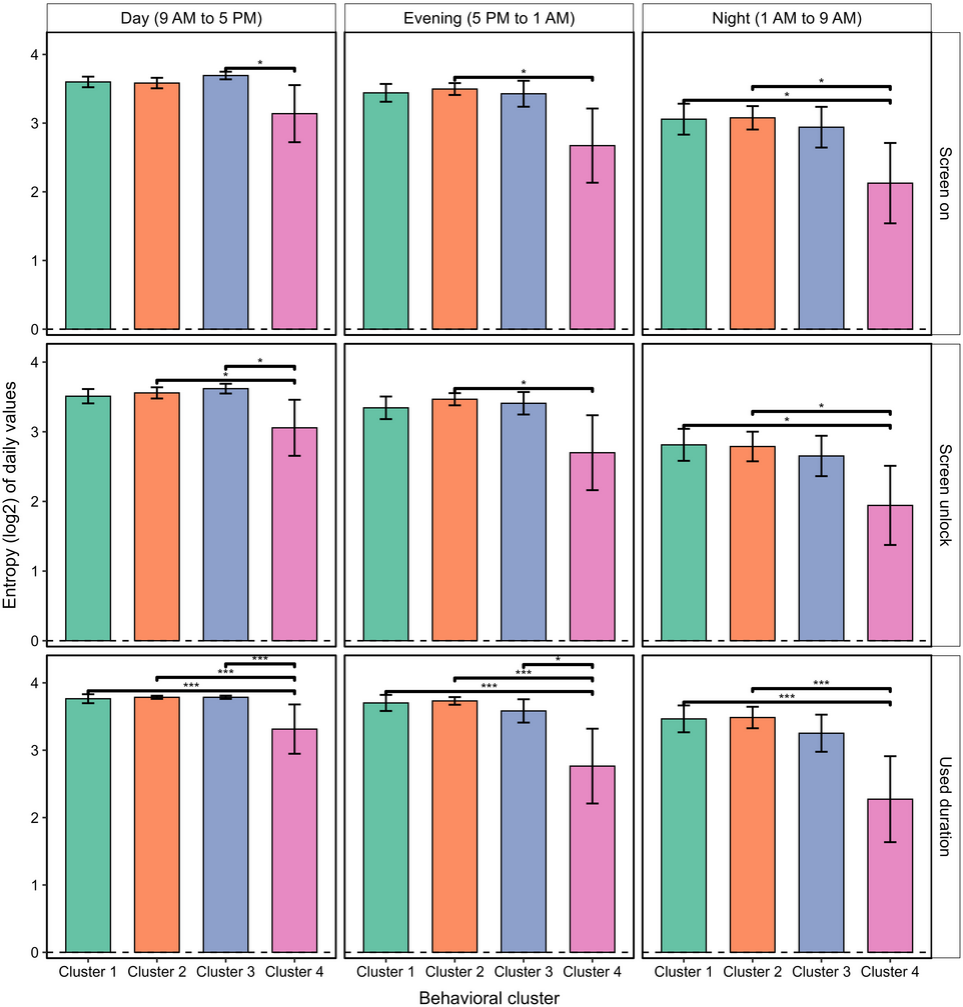
Comparisons of Shannon entropy of temporal usage metrics based on behavioral clusters. Bars represent the mean Shannon entropy of temporal metrics across individuals in each cluster, with error bars indicating 95% CIs. Bar colors indicate cluster 1 (green), cluster 2 (orange), cluster 3 (blue), and cluster 4 (pink). Asterisks indicate Bonferroni-adjusted pairwise Dunn test results: *P<.05, **P<.01, and ***P<.001. Only significance levels corresponding to the observed pairwise comparisons are displayed.

Notably, comparing entropy values revealed meaningful group differences. When entropy was computed to assess how much each metric’s within-individual variability was exhibited across 14 days, cluster 4 demonstrated significantly lower entropy in time-specific screen usage duration across all periods compared with clusters 1 and 2, suggesting more temporally structured behavior. By contrast, clusters 1 and 2 exhibited higher entropy values in screen usage by period, indicating relatively irregular usage patterns. A similar trend was observed in other screen-related metrics (eg, screen-on count and unlock count) between cluster 4 versus clusters 1 and 2, although significant differences emerged only in some periods (Figure 5). Table S8 in Multimedia Appendix 1 presents detailed statistical values. No consistent differences were found across clusters for other app usage–related or activity-related entropy metrics, except for some variables.

## Discussion

### Principal Findings

This study demonstrated that smartphone-derived behavioral data reported moderate performance in predicting short-term changes in depression and anxiety symptoms over a 2-week period. Simultaneously, we found that predictive performance diminished substantially when baseline Hamilton scores were removed, indicating that baseline symptom severity accounted for a large proportion of model performance. Additionally, subgroup analyses indicated no significant differences in performance across demographic groups, while clustering analysis revealed distinct behavioral patterns associated with symptom improvement, particularly those characterized by higher overall activity levels, greater engagement across multiple app usage categories, more daytime-focused usage, and greater temporal regularity.

### Prediction of Changes in HAM Scores Over 2 Weeks

We used a random forest model and evaluated its predictive ability for changes in depression (HAM-D) and anxiety (HAM-A) symptoms over 2 weeks, which demonstrated moderate predictive performance, particularly for HAM-D score changes (mean accuracy and mean AUROC of 0.70 [SD 0.02] and 0.74 [SD 0.02], respectively). The model performed relatively less effectively in predicting symptom deterioration (ie, increased scores), as reflected by the comparatively lower *F*_1_-scores (mean *F*_1_-scores: 0.60 [SD 0.10] and 0.61 [SD 0.09] for HAM-A and HAM-D, respectively); notably, only a small proportion of participants (14/95, 14.7%) demonstrated worsening symptoms, resulting in insufficient training examples for that outcome.

In the first secondary analysis, wherein the train-test split was applied before autoencoder training and repeated 20 times, performance metrics were comparable with our primary analysis, suggesting that the potential data leakage during the CAE training stage did not substantially inflate model performance. Moderate variability emerged across participant splits, indicating that the model is somewhat specific to the composition of the training and test data. Although this variability may reflect a degree of overfitting to particular data partitions, similar levels of variability were also observed when using a penalized logistic regression model instead of the random forest classifier; this observation suggests that the observed variability is more likely attributable to the modest sample size and differences in individual composition rather than model-specific overfitting. Nevertheless, the sensitivity to participant composition underscores the need for future studies with larger cohorts to further stabilize the embedding space.

A key result is that performance diminished substantially when baseline Hamilton scores were excluded. Mean accuracy reduced from 0.65 to 0.53 for HAM-A and from 0.70 to 0.47 for HAM-D, with corresponding reductions in the AUROC and *F*_1_-score, suggesting that the primary models relied heavily on baseline symptom severity. As nearly half of the participants remained in the unchanged category (HAM-A: 47/95, 49.5%; HAM-D: 45/95, 47.4%), the performance of models excluding baseline Hamilton scores should be interpreted against both equal probability chance and the majority-class baseline; from this perspective, passive smartphone-derived features without baseline symptom severity exhibited limited stand-alone predictive value in this predominantly nonclinical sample.

Several prior studies have reported encouraging performance in modeling symptom changes using smartphone-derived data. Ikaheimonen et al [15] reported an accuracy of 75% (95% CI 72%‐76%) in predicting changes in depressive symptom status; meanwhile, Kim et al [16] demonstrated a 3-fold validation accuracy of 76% in predicting antidepressant treatment response among adolescents. However, most prior studies developing prediction models of symptom changes have used clinical populations and binary definitions of symptom improvement (eg,≥40% reduction), whereas our study modeled the direction of symptom change in a predominantly nonclinical sample. Considering the generally low symptom severity and small magnitude of symptom change in our sample, passive data may have contained less symptom-related information than in clinical samples, potentially contributing to the relatively lower performance observed here. Although direct comparison with previous studies is limited owing to methodological differences, our findings underscore that the usefulness of passive digital phenotyping for symptom monitoring may depend on symptom range, sample composition, and outcome definition.

### Demographic Differences in Model Performance

Subgroup analyses, which compared the predictive model’s performance between the sex and age groups, revealed better performance among older participants than among younger participants and among males than among females. However, permutation tests—aiming to determine whether these differences truly reflected group characteristics—revealed no significant results. This result may be attributable to the study’s sample size and the participants’ relatively homogeneous age range (19‐54 years), potentially reducing the likelihood of a median-based dichotomization reflecting biologically meaningful age differences. Although significant performance differences were not identified between demographic groups, the subgroup-specific data characteristics revealed several noteworthy patterns. For instance, in age-group comparisons, the younger group exhibited greater interindividual variability in total smartphone usage, which may, in turn, have diminished the predictive performance observed in this group. Considering the small sample size, absence of significant permutation test results, and limited age range, these subgroup analyses should be interpreted as exploratory rather than confirmatory. Nevertheless, examining demographic variability remains crucial for digital phenotyping research, as prior studies have suggested that mobile phone–based mental health assessment algorithms perform differently across demographic groups [25,26].

### Behavioral Patterns Associated With Symptom Change

When individuals were grouped using clustering methods applied to autoencoder-compressed data, certain differences between clusters emerged in the raw cumulative values and entropy features. Participants in cluster 4—demonstrating the greatest reduction in HAM-D scores—exhibited higher overall behavioral metrics (including maps, productivity, and social apps, as well as daily moving distance measured by smartphone sensors) and day-focused, regular patterns in usage of smartphones. The observed patterns broadly align with prior findings suggesting that physical and social activities help prevent or mitigate depressive symptoms [27,28].

Additionally, concerning smartphone use’s temporal distribution throughout the day, cluster 4 exhibited a “day-focused” usage pattern, characterized by concentrated use during the daytime (9 AM to 5 PM) and relatively lower use in the evening (5 PM to 1 AM) and nighttime (1 AM to 9 AM). By contrast, clusters 1 and 2—exhibiting a slight increase or a minimal decrease in HAM-D score—demonstrated more evenly distributed usage across all time periods, reflecting a “throughout-the-day” pattern. Moreover, their entropy values for time-specific usage indicators were significantly higher, indicating more irregular temporal patterns of smartphone use. These findings align with the literature linking irregular daily routines and disrupted circadian rhythms to adverse mental health outcomes [29,30]. Notably, previous studies concerning the relationship between temporal smartphone usage patterns and mood have yielded inconsistent results [25,31,32], likely reflecting the data’s inherently complex and multifactorial nature; by contrast, our clustering approach highlights that combinations of behavioral features, rather than single indicators, may better capture meaningful differences in mental health trajectories.

Nevertheless, significant differences across clusters were primarily observed in depressive symptoms and were less pronounced for anxiety. This is partially attributable to our sample’s characteristics, as participants were drawn from a nonclinical sample, with most individuals presenting relatively low levels of anxiety and depression symptoms. Furthermore, prior studies have demonstrated that the presentation of anxiety symptoms in smartphone-derived features exhibits greater variation across individuals [13] and that the types of smartphone-derived features associated with depressive symptom severity differ from those associated with anxiety [5]. This may partially explain why changes in depressive symptoms—typically associated with sustained daily routines and behavioral regularity—exhibited clearer differentiation across clusters.

### Limitations and Future Directions

This study has several limitations. First, several methodological features of the primary analytical approach, which motivated the secondary analyses, should be considered when interpreting the findings. The autoencoder was initially trained using the full dataset, potentially raising concerns regarding data leakage and the potential overestimation of model performance. We addressed this issue by conducting the first secondary analysis, which involved applying train-test separation before autoencoder training and yielded comparable results. Nevertheless, this issue should be considered when interpreting the main findings. In another secondary analysis, the substantial decline in model performance following the exclusion of baseline Hamilton scores indicated that passive smartphone-derived variables provided only modest incremental predictive value beyond initial symptom severity. Thus, this study’s findings should not be interpreted as supporting passive smartphone monitoring as a stand-alone approach for symptom trajectory classification.

Second, the latent representations extracted from the autoencoder were further reduced using PCA before being input into the predictive model. This step—although necessary to mitigate dimensionality relative to the sample size—may have resulted in some loss of information, particularly concerning temporal dynamics embedded within the latent space.

Third, the study sample largely comprised individuals without clinically significant psychiatric symptoms, fostering relatively small absolute changes in HAM-A and HAM-D scores over the 2-week period. Although the model predicted these changes above chance level, the clinical meaningfulness of such small fluctuations remains uncertain.

Fourth, the overall sample size was small for a machine learning study, particularly considering the 3-class outcome structure and the additional subgroup and clustering analyses. Accordingly, the subgroup and clustering findings should be regarded as exploratory and hypothesis-generating. Cluster 4, which exhibited the greatest improvement in depressive symptoms, included the smallest number of participants; consequently, caution is warranted in interpreting the clustering results.

Furthermore, participants with missing data on even a single day during the 14-day monitoring period were excluded from the analyses, potentially introducing bias and limiting the findings’ generalizability. Most exclusions were attributable to a lack of consent for app usage tracking. Participants who were excluded from the analyses were typically older on average and exhibited slightly lower baseline HAM-A and HAM-D scores. Finally, only those participants using Android smartphones were included owing to app compatibility constraints, potentially limiting generalizability, as smartphone usage patterns and user characteristics differ across device platforms.

Future studies should use larger and more symptomatically heterogeneous samples, evaluate external validation, and more explicitly compare models based on passive data alone, clinical baseline variables alone, and their combination. Such designs would help clarify the conditions under which passive smartphone-derived data provide clinically meaningful added value.

### Conclusions

This study highlights that passive smartphone-derived behavioral data may reflect short-term fluctuations in depression and anxiety symptoms; however, their independent predictive value beyond baseline symptom severity was modest in this predominantly nonclinical sample. These findings underscore that passive smartphone data may be less informative, particularly among individuals with subclinical symptoms, thereby warranting careful processing and interpretation. Meanwhile, the identification of behavioral phenotypes associated with depressive symptom changes emphasizes that passive digital data may still enhance our understanding of how mental health–related behavioral patterns manifest in everyday life. Even when their independent predictive value is limited, passive data may provide descriptive and hypothesis-generating insights into behaviorally meaningful subtypes.

## Supplementary material

10.2196/88083 Multimedia Appendix 1 Details of collected data and derived features, including supplementary tables and figures.

10.2196/88083 Multimedia Appendix 2 Anonymized derived feature data used as input for the convolutional autoencoder processing.

10.2196/88083 Multimedia Appendix 3 Python code used to process smartphone-derived metrics with the convolutional autoencoder.

10.2196/88083 Multimedia Appendix 4 Processed values generated by the Python code provided in Multimedia Appendix 3.

## Abbreviations

AMC: Asan Medical Center
CAE: convolutional autoencoder
HAM-A: Hamilton Anxiety Rating Scale
HAM-D: Hamilton Depression Rating Scale
PCA: principal component analysis
