# Supplementary material for: Modeling Short-Term Symptom Changes and Behavioral Subtypes of Depression and Anxiety in the General Population: Observational Study Using Smartphone Data
Source: JMIR Form Res. 2026 Jul 14;10:e88083. doi: 10.2196/88083 (PMC13367947; doi:10.2196/88083)

Multimedia Appendix 1

# **Collected data and derived features**

**Raw data**

**Physical activity and environmental context metrics**

- **Moved distance**: change in GPS location measured at 10-min intervals (m)
- **Light**: average light amplitude during the hour (lux)

**Screen usage data**

- **Screen on**: total count of screen-on events aggregated within each hour
- **Screen unlock**: total count of screen unlock events aggregated within each hour
- **Used duration**: total duration of screen-on time aggregated within each hour (seconds)

**App usage logs**

- **App usage**: continuous logs including app name, app usage start time, and app usage end time

**Derived features employed for autoencoder training**

All data were extracted for a 2-week period (336 hours) from the start of data collection. Subsequently, the data were aggregated into 24-hour intervals to define Days 1 through 14.

All features were standardized within each individual using z-scores based on the 14-day observations.

**Physical activity and environmental context metrics**

- **Moved distance**: for each Day, the sum of GPS location changes measured at 10-minute intervals (m)
- **Light**: for each Day, the sum of hourly averaged ambient light levels (lux)

**Screen usage data**

For each Day, the time was divided into three periods, resulting in the following nine variables:

- **Screen on (day)**: for each day, the total number of screen-on events between 09:00 (inclusive) and 17:00 (exclusive)
- **Screen unlock (day)**: for each day, the total number of screen unlock events between 09:00 and 17:00
- **Used duration (day)**: for each day, the total screen-on duration between 09:00 and 17:00 (seconds)

Using the same approach, the following features were generated for the evening (17:00–01:00) and night (01:00–09:00) periods:

- **Screen on (evening)**
- **Screen unlock (evening)**
- **Used duration (evening)**
- **Screen on (night)**
- **Screen unlock (night)**
- **Used duration (night)**

**App usage logs**

Apps were categorized into nine groups based on the categories provided by the Google Play Store, and the daily usage duration (seconds) for each category was computed:

- **AppCategory_Audio apps**: apps in the *Music & Audio* category
- **AppCategory_Game apps**: apps in the *Game* category
- **AppCategory_Image apps**: apps in the *Photography* category
- **AppCategory_Maps apps**: apps in the *Maps & Navigation* category
- **AppCategory_News apps**: apps in the *News & Magazines* category
- **AppCategory_Productivity apps**: apps in the *Productivity* category
- **AppCategory_Social apps**: apps in the *Social* category
- **AppCategory_Video apps**: apps in the *Video Players & Editors* and *Entertainment* categories
- **AppCategory_Undefined apps**: apps not falling into any of the above categories

Multimedia Appendix 2 provides the anonymized derived feature.

# **Table S1. Comparison of Demographics and HAM-D/HAM-A Scores between Included and Excluded Participants**

|  | Included (n = 95) | Excluded (n = 63) | χ² | *df* | *P* |
| --- | --- | --- | --- | --- | --- |
|  |  |  |  |  |  |
| Age, mean (SD) ^a, b^ | 30.4 (9.5) | 33.5 (9.7) | 5.40 | 1 | .02 |
| Male, n (%) ^b, c^ | 51 (53.7) | 33 (54.1) | 0 | 1 | 1 |
| Baseline total scores ^a^ |  |  |  |  |  |
| HAM-A, mean (SD) | 2.48 (3.63) | 1.67 (3.25) | 4.95 | 1 | .03 |
| HAM-D, mean (SD) | 2.41 (1.49) | 1.49 (2.97) | 6.02 | 1 | .01 |
| Week 2 total scores ^a^ |  |  |  |  |  |
| HAM-A, mean (SD) | 1.92 (3.65) | 1.16 (2.43) | 2.08 | 1 | .15 |
| HAM-D, mean (SD) | 1.99 (3.78) | 1.25 (2.68) | 3.13 | 1 | .08 |

HAM-A, Hamilton Anxiety Rating Scale; HAM-D, Hamilton Depression Rating Scale

^a^ Kruskal–Wallis rank sum test

^b^ Two participants had missing values for both age and sex and were excluded from analyses involving these variables.

^c^ Chi-square test

# **Table S2. Additional Participant Demographics**

| Variable | Value |
| --- | --- |
| Total | 95 |
| Age, mean (SD) | 30.36 (9.50) |
| Sex, n (%) |  |
| Male | 51 (54) |
| Female | 44 (46) |
| Occupation, n (%) |  |
| Student (undergraduate/graduate) | 53 (56) |
| Office worker | 14 (15) |
| Unemployed | 2 (2) |
| Physician | 2 (2) |
| No response | 24 (25) |
| Prior psychiatric diagnosis, n (%) |  |
| No diagnosis | 94 (99) |
| Major depressive disorder | 1 (1) |

# **Table S3a. Changes in HAM-A Scores Based on Demographic Characteristics**

|  | Number of participants (%) | HAM-A Score Change^a^, mean (SD) | Test statistic | *df* | *P* |
| --- | --- | --- | --- | --- | --- |
|  |  |  |  |  |  |
| Total | 95 | -0.57 (2.32) |  |  |  |
| Sex^b^ |  |  |  |  |  |
| Male | 51 (54) | -1.12 (1.86) | χ² = 6.59 | 1 | .01 |
| Female | 44 (46) | 0.07 (2.64) |  |  |  |
| Age group^b^ |  |  |  |  |  |
| Age ≤27 | 48 (50) | -0.33 (2.26) | χ² = 0.21 | 1 | .64 |
| Age >27 | 47 (50) | -0.81 (2.37) |  |  |  |
| Age (continuous)^c^ |  |  | ρ = -0.01 | - | .94 |

HAM-A, Hamilton Anxiety Rating Scale; HAM-D, Hamilton Depression Rating Scale

^a^ Calculated as (Week 2 − Baseline) for HAM-A and HAM-D total scores

^b^ Kruskal–Wallis rank sum test

^c^ Spearman correlation test

# **Table S3b. Changes in HAM-D Scores Based on Demographic Characteristics**

|  | Number of participants (%) | HAM-D Score Change^a^, mean (SD) | Test statistic | *df* | *P* |
| --- | --- | --- | --- | --- | --- |
|  |  |  |  |  |  |
| Total | 95 | -0.42 (2.06) |  |  |  |
| Sex^b^ |  |  |  |  |  |
| Male | 51 (54) | -0.80 (1.93) | χ² = 2.08 | 1 | .15 |
| Female | 44 (46) | 0.02 (2.13) |  |  |  |
| Age group^b^ |  |  |  |  |  |
| Age ≤27 | 48 (50) | -0.56 (2.29) | χ² = 0.69 | 1 | .40 |
| Age >27 | 47 (50) | -0.28 (1.80) |  |  |  |
| Age (continuous)^c^ |  |  | ρ = 0.08 | - | .46 |

HAM-A, Hamilton Anxiety Rating Scale; HAM-D, Hamilton Depression Rating Scale

^a^ Calculated as (Week 2 − Baseline) for HAM-A and HAM-D total scores

^b^ Kruskal–Wallis rank sum test

^c^ Spearman correlation test

# **Table S4. Group Comparisons of Smartphone Usage Metrics Based on Age and Sex (Kruskal–Wallis Test Results)**

| Variable | | *P* | | Difference of direction by rank | | Mean value comparison | |
| --- | --- | --- | --- | --- | --- | --- | --- |
| Age group comparisons | |  | |  | |  | |
| Used duration total | | .01 | | old > young | | young > old | |
| Social apps | | < .001 | | young > old | | young > old | |
| Audio apps | | < .001 | | young > old | | young > old | |
| Screen unlock (night) | | < .001 | | old > young | | young > old | |
| Video apps | | < .001 | | young > old | | old > young | |
| Screen on (night) | | < .001 | | old > young | | young > old | |
| Map apps | | < .001 | | young > old | | young > old | |
| Used duration (night) | | < .001 | | old > young | | young > old | |
| Image apps | | < .001 | | young > old | | young > old | |
| News apps | | < .001 | | young > old | | young > old | |
| Game apps | | < .001 | | young > old | | old > young | |
| Moved distance | | .01 | | young > old | | young > old | |
| Screen on (evening) | | .01 | | young > old | | young > old | |
| Screen unlock (day) | | .02 | | old > young | | young > old | |
| Productivity apps | | .03 | | old > young | | old > young | |
| Used duration (day) | | .053 | | old > young | | young > old | |
| Screen unlock (evening) | | .07 | | young > old | | young > old | |
| Light | | .42 | | old > young | | old > young | |
| Used duration (evening) | | .49 | | old > young | | young > old | |
| Screen on (day) | | .55 | | old > young | | young > old | |
| Undefined apps | | .76 | | young > old | | old > young | |
| Sex group comparisons | |  | |  | |  | |
| Used duration total | | < .001 | | male > female | | male > female | |
| Video apps | | < .001 | | male > female | | male > female | |
| Screen unlock (evening) | | < .001 | | male > female | | male > female | |
| Moved distance | | < .001 | | male > female | | male > female | |
| Screen on (evening) | | < .001 | | male > female | | male > female | |
| Screen unlock (day) | | < .001 | | male > female | | male > female | |
| Undefined apps | | < .001 | | female > male | | female > male | |
| Used duration (evening) | | < .001 | | male > female | | female > male | |
| Productivity apps | | < .001 | | female > male | | female > male | |
| Screen on (day) | | < .001 | | male > female | | male > female | |
| Audio apps | | < .001 | | male > female | | female > male | |
| Used duration (day) | | < .001 | | male > female | | male > female | |
| Game apps | | < .001 | | male > female | | male > female | |
| News apps | | < .001 | | female > male | | female > male | |
| Image apps | | < .001 | | female > male | | female > male | |
| Light | | < .001 | | male > female | | male > female | |
| Used duration (night) | | .02 | | male > female | | male > female | |
| Screen on (night) | | .13 | | male > female | | male > female | |
| Map apps | | .17 | | male > female | | male > female | |
| Screen unlock (night) | | .27 | | male > female | | male > female | |
| Social apps | | .55 | | female > male | | male > female | |

# **Table S5. Results of Secondary Analysis With Train-Test Split Before CAE Training Across 20 Random Seeds**

|  | HAM-A | | | HAM-D | | |
| --- | --- | --- | --- | --- | --- | --- |
| Splits seed | Accuracy | AUC | *F*_1_ | Accuracy | AUC | *F*_1_ |
| 1 | 0.58 | 0.66 | 0.54 | 0.74 | 0.80 | 0.77 |
| 2 | 0.79 | 0.72 | 0.86 | 0.68 | 0.77 | 0.74 |
| 3 | 0.89 | 0.60 | 0.92 | 0.84 | 0.75 | 0.83 |
| 4 | 0.74 | 0.83 | 0.71 | 0.84 | 0.93 | 0.83 |
| 5 | 0.58 | 0.73 | 0.65 | 0.68 | 0.59 | 0.75 |
| 6 | 0.74 | 0.87 | 0.75 | 0.74 | 0.87 | 0.70 |
| 7 | 0.63 | 0.83 | 0.65 | 0.47 | 0.67 | 0.54 |
| 8 | 0.58 | 0.64 | 0.65 | 0.58 | 0.77 | 0.67 |
| 9 | 0.68 | 0.78 | 0.68 | 0.58 | 0.61 | 0.66 |
| 10 | 0.63 | 0.60 | 0.66 | 0.53 | 0.61 | 0.60 |
| 11 | 0.63 | 0.60 | 0.68 | 0.68 | 0.59 | 0.72 |
| 12 | 0.74 | 0.84 | 0.75 | 0.74 | 0.74 | 0.67 |
| 13 | 0.74 | 0.79 | 0.80 | 0.68 | 0.75 | 0.79 |
| 14 | 0.47 | 0.52 | 0.47 | 0.53 | 0.56 | 0.53 |
| 15 | 0.74 | 0.75 | 0.76 | 0.74 | 0.78 | 0.76 |
| 16 | 0.63 | 0.76 | 0.73 | 0.74 | 0.72 | 0.86 |
| 17 | 0.74 | 0.46 | 0.76 | 0.68 | 0.69 | 0.76 |
| 18 | 0.68 | 0.89 | 0.74 | 0.53 | 0.73 | 0.62 |
| 19 | 0.74 | 0.82 | 0.80 | 0.68 | 0.61 | 0.70 |
| 20 | 0.74 | 0.68 | 0.71 | 0.79 | 0.87 | 0.82 |
| Mean across splits (SD) | 0.68 (0.09) | 0.72 (0.12) | 0.71 (0.10) | 0.67 (0.11) | 0.72 (0.10) | 0.72 (0.09) |

CAE, convolutional autoencoder; HAM-A, Hamilton Anxiety Rating Scale; HAM-D, Hamilton Depression Rating Scale

# **Table S6. Secondary Analysis Results Using Penalized Logistic Regression With Train-Test Split Before CAE Training (20 Random Seeds)**

|  | HAM-A | | | HAM-D | | |
| --- | --- | --- | --- | --- | --- | --- |
| Splits seed | Accuracy | AUC | *F*_1_ | Accuracy | AUC | *F*_1_ |
| 1 | 0.68 | 0.47 | 0.63 | 0.63 | 0.52 | 0.68 |
| 2 | 0.58 | 0.67 | 0.60 | 0.68 | 0.58 | 0.73 |
| 3 | 0.47 | 0.54 | 0.51 | 0.47 | 0.66 | 0.48 |
| 4 | 0.47 | 0.73 | 0.45 | 0.74 | 0.76 | 0.74 |
| 5 | 0.58 | 0.72 | 0.66 | 0.58 | 0.65 | 0.71 |
| 6 | 0.47 | 0.53 | 0.50 | 0.58 | 0.68 | 0.65 |
| 7 | 0.74 | 0.73 | 0.82 | 0.63 | 0.80 | 0.70 |
| 8 | 0.53 | 0.72 | 0.47 | 0.74 | 0.75 | 0.75 |
| 9 | 0.79 | 0.89 | 0.72 | 0.84 | 0.82 | 0.88 |
| 10 | 0.63 | 0.62 | 0.63 | 0.47 | 0.71 | 0.52 |
| 11 | 0.74 | 0.64 | 0.75 | 0.79 | 0.67 | 0.77 |
| 12 | 0.63 | 0.93 | 0.77 | 0.89 | 0.88 | 0.88 |
| 13 | 0.47 | 0.62 | 0.38 | 0.74 | 0.58 | 0.80 |
| 14 | 0.63 | 0.77 | 0.62 | 0.74 | 0.69 | 0.71 |
| 15 | 0.68 | 0.45 | 0.72 | 0.47 | 0.70 | 0.54 |
| 16 | 0.63 | 0.58 | 0.68 | 0.63 | 0.74 | 0.73 |
| 17 | 0.47 | 0.56 | 0.49 | 0.47 | 0.66 | 0.56 |
| 18 | 0.53 | 0.54 | 0.46 | 0.53 | 0.64 | 0.58 |
| 19 | 0.74 | 0.64 | 0.78 | 0.63 | 0.56 | 0.72 |
| 20 | 0.58 | 0.75 | 0.57 | 0.63 | 0.77 | 0.69 |
| Mean across splits (SD) | 0.61 (0.10) | 0.65 (0.13) | 0.62 (0.12) | 0.64 (0.12) | 0.69 (0.09) | 0.69 (0.11) |

CAE, convolutional autoencoder; HAM-A, Hamilton Anxiety Rating Scale; HAM-D, Hamilton Depression Rating Scale

# **Table S7. Secondary Analysis Results Excluding Baseline Hamilton Scores From Predictors With Train-Test Split Before CAE Training (20 Random Seeds)**

|  | HAM-A | | | HAM-D | | |
| --- | --- | --- | --- | --- | --- | --- |
| Splits seed | Accuracy | AUC | *F*_1_ | Accuracy | AUC | *F*_1_ |
| 1 | 0.43 | 0.38 | 0.40 | 0.52 | 0.57 | 0.48 |
| 2 | 0.53 | 0.61 | 0.47 | 0.45 | 0.52 | 0.41 |
| 3 | 0.50 | 0.58 | 0.55 | 0.32 | 0.51 | 0.35 |
| 4 | 0.42 | 0.62 | 0.37 | 0.49 | 0.59 | 0.47 |
| 5 | 0.46 | 0.63 | 0.50 | 0.59 | 0.68 | 0.69 |
| 6 | 0.42 | 0.34 | 0.44 | 0.55 | 0.52 | 0.62 |
| 7 | 0.49 | 0.70 | 0.55 | 0.35 | 0.59 | 0.40 |
| 8 | 0.54 | 0.65 | 0.56 | 0.49 | 0.52 | 0.49 |
| 9 | 0.64 | 0.73 | 0.59 | 0.57 | 0.75 | 0.57 |
| 10 | 0.63 | 0.60 | 0.64 | 0.46 | 0.69 | 0.46 |
| 11 | 0.72 | 0.53 | 0.73 | 0.59 | 0.60 | 0.60 |
| 12 | 0.52 | 0.49 | 0.48 | 0.49 | 0.42 | 0.42 |
| 13 | 0.47 | 0.63 | 0.54 | 0.56 | 0.51 | 0.61 |
| 14 | 0.54 | 0.62 | 0.50 | 0.56 | 0.74 | 0.60 |
| 15 | 0.50 | 0.71 | 0.48 | 0.24 | 0.44 | 0.28 |
| 16 | 0.57 | 0.54 | 0.61 | 0.43 | 0.64 | 0.49 |
| 17 | 0.60 | 0.73 | 0.61 | 0.37 | 0.55 | 0.41 |
| 18 | 0.53 | 0.54 | 0.48 | 0.34 | 0.50 | 0.36 |
| 19 | 0.53 | 0.57 | 0.52 | 0.44 | 0.45 | 0.44 |
| 20 | 0.58 | 0.66 | 0.62 | 0.51 | 0.67 | 0.56 |
| Mean across splits (SD) | 0.53 (0.08) | 0.59 (0.11) | 0.53 (0.10) | 0.47 (0.10) | 0.57 (0.09) | 0.49 (0.10) |

CAE, convolutional autoencoder; HAM-A, Hamilton Anxiety Rating Scale; HAM-D, Hamilton Depression Rating Scale

# **Table S8. Post Hoc Analysis of Shannon Entropy Comparisons of Temporal Usage Metrics Based on Behavioral Clusters**

| Variable | Time | Comparison | Z | Adjusted P |
| --- | --- | --- | --- | --- |
| Screen on | Day | Clusters 1–2 | 0.133 | 1 |
| Screen on | Day | Clusters 1–3 | -1.63 | .61 |
| Screen on | Day | Clusters 2–3 | -1.85 | .38 |
| Screen on | Day | Clusters 1–4 | 1.64 | .61 |
| Screen on | Day | Clusters 2–4 | 1.61 | .65 |
| Screen on | Day | Clusters 3–4 | 2.95 | .02 |
| Screen on | Evening | Clusters 1–2 | -0.446 | 1 |
| Screen on | Evening | Clusters 1–3 | -0.219 | 1 |
| Screen on | Evening | Clusters 2–3 | 0.18 | 1 |
| Screen on | Evening | Clusters 1–4 | 2.48 | .08 |
| Screen on | Evening | Clusters 2–4 | 2.96 | .02 |
| Screen on | Evening | Clusters 3–4 | 2.54 | .07 |
| Screen on | Night | Clusters 1–2 | -0.00833 | 1 |
| Screen on | Night | Clusters 1–3 | 0.528 | 1 |
| Screen on | Night | Clusters 2–3 | 0.567 | 1 |
| Screen on | Night | Clusters 1–4 | 2.89 | .02 |
| Screen on | Night | Clusters 2–4 | 3.03 | .01 |
| Screen on | Night | Clusters 3–4 | 2.3 | .13 |
| Screen unlock | Day | Clusters 1–2 | -0.663 | 1 |
| Screen unlock | Day | Clusters 1–3 | -1.23 | 1 |
| Screen unlock | Day | Clusters 2–3 | -0.689 | 1 |
| Screen unlock | Day | Clusters 1–4 | 2.09 | .22 |
| Screen unlock | Day | Clusters 2–4 | 2.74 | .04 |
| Screen unlock | Day | Clusters 3–4 | 3.04 | .01 |
| Screen unlock | Evening | Clusters 1–2 | -0.882 | 1 |
| Screen unlock | Evening | Clusters 1–3 | -0.429 | 1 |
| Screen unlock | Evening | Clusters 2–3 | 0.359 | 1 |
| Screen unlock | Evening | Clusters 1–4 | 2.24 | .15 |
| Screen unlock | Evening | Clusters 2–4 | 3.07 | .01 |
| Screen unlock | Evening | Clusters 3–4 | 2.5 | .07 |
| Screen unlock | Night | Clusters 1–2 | 0.0682 | 1 |
| Screen unlock | Night | Clusters 1–3 | 0.681 | 1 |
| Screen unlock | Night | Clusters 2–3 | 0.66 | 1 |
| Screen unlock | Night | Clusters 1–4 | 2.75 | .04 |
| Screen unlock | Night | Clusters 2–4 | 2.83 | .03 |
| Screen unlock | Night | Clusters 3–4 | 2.04 | .25 |
| Used duration | Day | Clusters 1–2 | -0.0494 | 1 |
| Used duration | Day | Clusters 1–3 | 0.193 | 1 |
| Used duration | Day | Clusters 2–3 | 0.25 | 1 |
| Used duration | Day | Clusters 1–4 | 4.27 | <.001 |
| Used duration | Day | Clusters 2–4 | 4.52 | <.001 |
| Used duration | Day | Clusters 3–4 | 3.9 | <.001 |
| Used duration | Evening | Clusters 1–2 | -0.167 | 1 |
| Used duration | Evening | Clusters 1–3 | 1.49 | .82 |
| Used duration | Evening | Clusters 2–3 | 1.74 | .50 |
| Used duration | Evening | Clusters 1–4 | 4.52 | <.001 |
| Used duration | Evening | Clusters 2–4 | 4.87 | <.001 |
| Used duration | Evening | Clusters 3–4 | 3.03 | .01 |
| Used duration | Night | Clusters 1–2 | -0.19 | 1 |
| Used duration | Night | Clusters 1–3 | 1.3 | 1 |
| Used duration | Night | Clusters 2–3 | 1.56 | .71 |
| Used duration | Night | Clusters 1–4 | 3.78 | <.001 |
| Used duration | Night | Clusters 2–4 | 4.12 | <.001 |
| Used duration | Night | Clusters 3–4 | 2.48 | .08 |

# **Figure S1. Distribution of Changes in HAM-A and HAM-D Scores
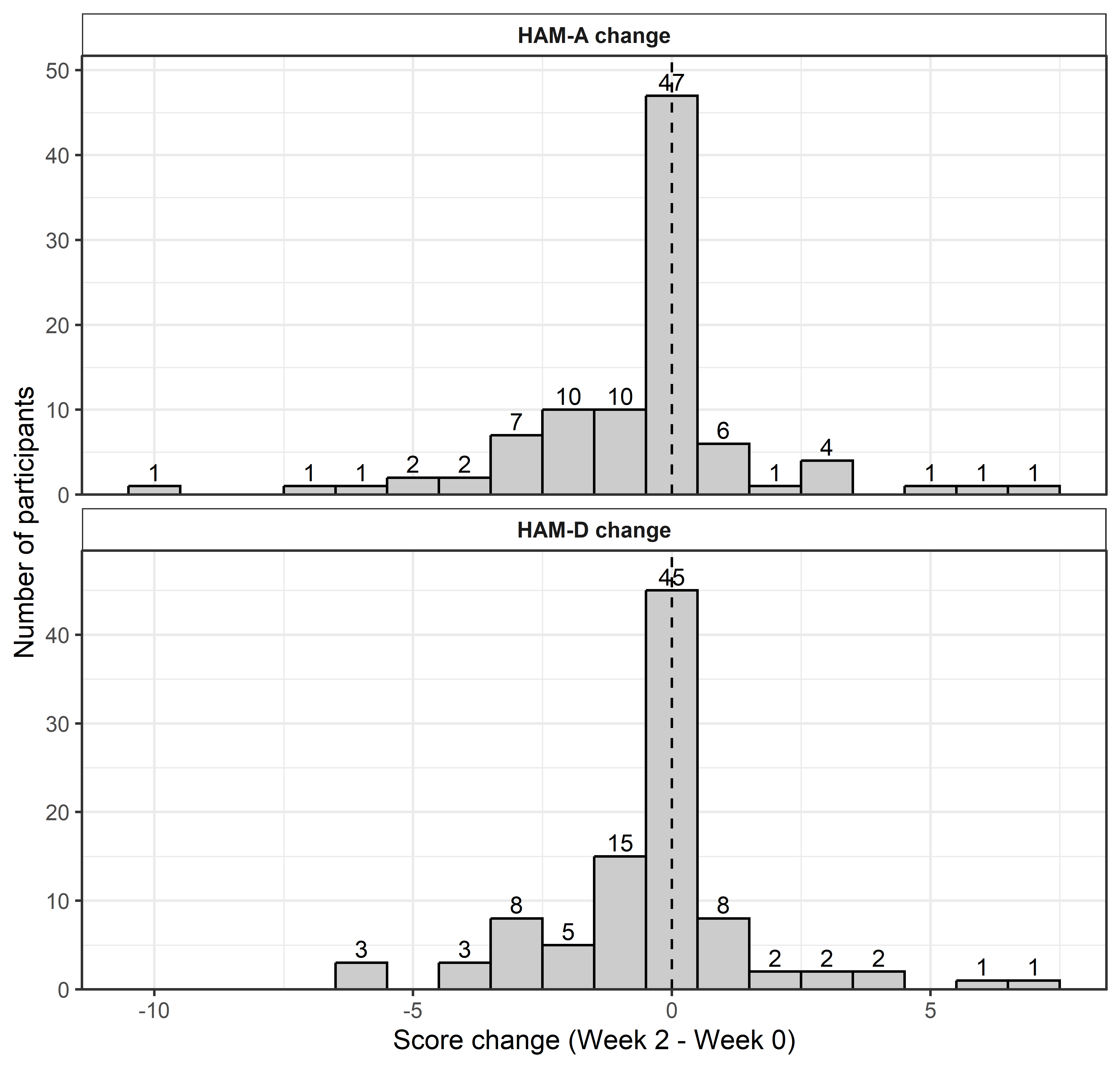
**

# **Figure S2. Distribution of Total Smartphone Usage Over 14 Days Based on Age Group** Distribution of total smartphone usage over the 14-day observation period according to age group (Younger versus Older, defined by median split). Boxes represent the interquartile range (IQR), the horizontal line within each box indicates the median, and individual points represent participants.


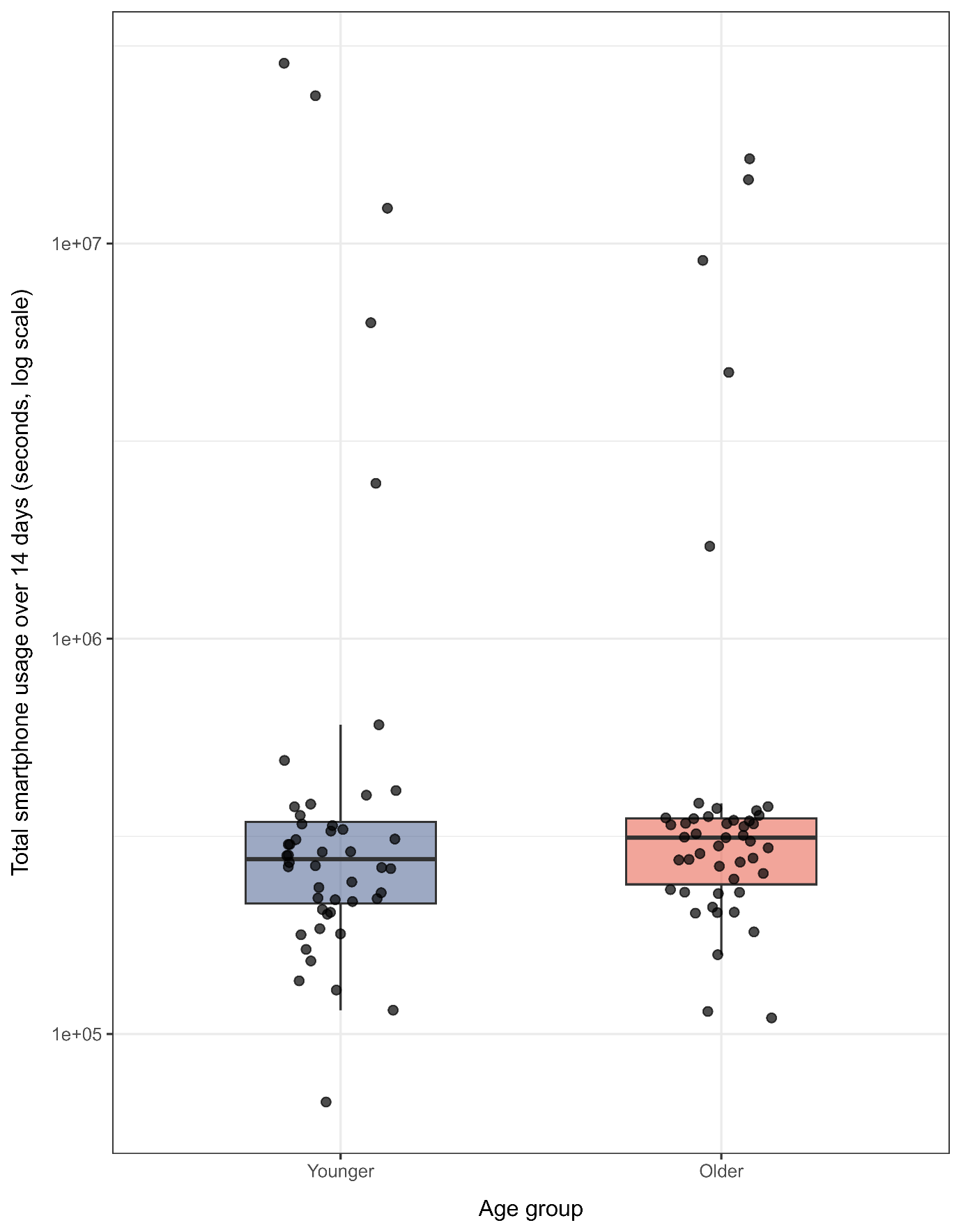


# **Figure S3. Distribution of Total Smartphone Usage Over 14 Days Based on Sex Group** Distribution of total smartphone usage over the 14-day observation period according to sex. Boxes represent the interquartile range (IQR), the horizontal line within each box indicates the median, and individual points represent participants.


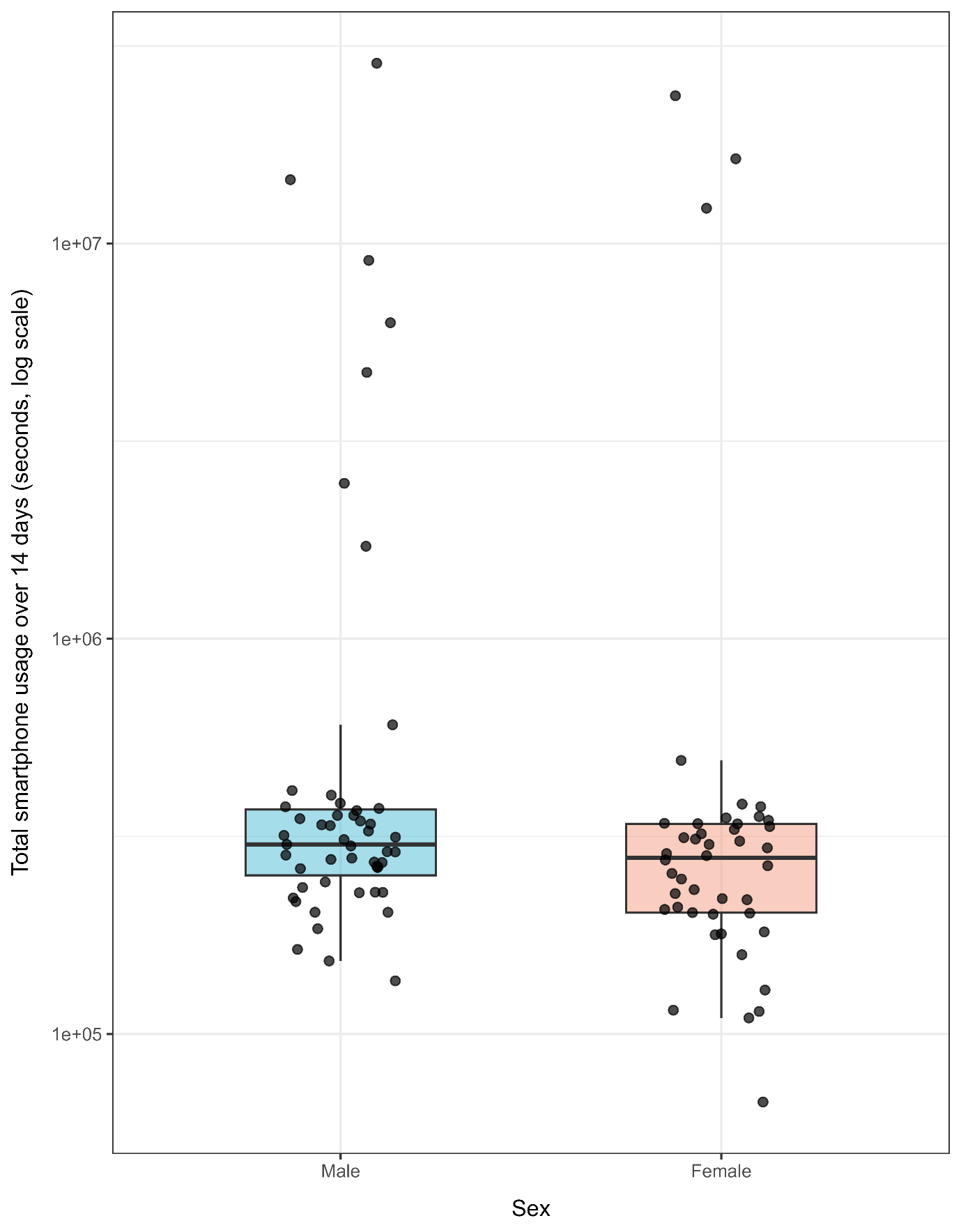

Supplement: Multimedia Appendix 1 [file formative-v10-e88083-s001.docx]
