# Supplementary material for: Modeling Short-Term Symptom Changes and Behavioral Subtypes of Depression and Anxiety in the General Population: Observational Study Using Smartphone Data
Source: JMIR Form Res. 2026 Jul 14;10:e88083. doi: 10.2196/88083 (PMC13367947; doi:10.2196/88083)
Supplement: Multimedia Appendix 3 [file formative-v10-e88083-s003.pdf]

main_individual_norm-

September 20, 2025

[36]:
```
import csv
import glob
import numpy as np
import pandas as pd
import tensorflow as tf
import matplotlib.pyplot as plt

from scipy import stats
from models import *
```

1 Data Preprocessing

[37]:
```
raw_data = np.genfromtxt("data/app_screen_data_2_mapped_filtered.csv",
                         delimiter=',',
                        usecols=(0, 2, 3, 4, 5, 6, 7, 8, 9, 10, 11, 12, 13, 14,␣
 ↪15, 16, 17, 18, 19, 21, 22))

index = raw_data[1:, 0:1]  # userid
value = np.concatenate((index, raw_data[1:, 1:]), axis=1)


X = []
userid_list = []
for userid in np.unique(index):
    user_data = value[np.where(value[:, 0] == userid)][:, 1:]
    X.append(user_data)
    userid_list.append(userid)

Sliding_X = []
uid_track = []
column_size = 7
user_minmax_list = []

for user_X, userid in zip(X, userid_list):
    user_min = np.min(user_X, axis=0, keepdims=True)
    user_max = np.max(user_X, axis=0, keepdims=True)
    user_mean = np.mean(user_X, axis=0, keepdims=True)
```

```
    user_diff = user_max - user_min
    user_diff[user_diff == 0] = 1e-8

    i = 0
    while i + column_size <= user_X.shape[0]:
        Sliding_X.append(user_X[i:i+column_size, :])
        user_minmax_list.append((user_min, user_diff, user_mean))
        uid_track.append(int(userid))
        i += 1

Preprocessed_X = []
uid_idx = 0
for window in Sliding_X:
    nonzero_data = window[~np.isnan(window).any(axis=1)]
    nonzero_data = nonzero_data[np.all(nonzero_data != -1, axis=1)]

    if nonzero_data.shape[0] == 0:
        mean = prev_mean
    else:
        mean = np.mean(nonzero_data, axis=0)[None, :]
        prev_mean = mean

    user_min, user_diff, user_mean = user_minmax_list[uid_idx]
    uid_idx += 1

    userX = np.array([])
    first = True
    for row in window:
        row = row.reshape(-1, len(row))
        if np.any(row[0, :] == -1, axis=0):
            minus_index = np.argwhere(row == -1)
            row[0, minus_index] = mean[0, minus_index]


        row_norm = (row - user_min) / user_diff
        scale_weight = 0
        row = scale_weight * row + (1 - scale_weight) * row_norm

        if first:
            first = False
            userX = row
        else:
            userX = np.concatenate((userX, row), axis=0)

    Preprocessed_X.append(userX)

Sliding_X = np.array(Preprocessed_X)
```

```
Sliding_X = Sliding_X.reshape(-1, 7, 20, 1)
```

2 Convolutional Autoencoder

[38]:

```
class RevisedConvolutionalAutoencoder(object):
    def __init__(self, filter_size, filter_num, encoded_size, learning_rate):
        x = tf.placeholder(tf.float32, shape=[None, 7, 20, 1])

        # encoder part
        conv1 = Convolution2D([filter_size, 20, 1, filter_num],␣
↪padding='VALID', activation=tf.nn.relu, scope='conv_1')(x)
        unfold = Unfold(scope='unfold')(conv1)
        encoded = FullyConnected(encoded_size, activation=tf.nn.relu,␣
↪scope='encode')(unfold)

        # decoder part
        decoded = FullyConnected((7 - filter_size + 1) * 1 * filter_num,␣
↪activation=tf.nn.relu, scope='decode')(encoded)
        fold = Fold([-1, (7 - filter_size + 1), 1, filter_num],␣
↪scope='fold')(decoded)
        reconstruction = DeConvolution2D([filter_size, 20, 1, filter_num],
                                         padding='VALID',
                                         output_shape=tf.shape(x),
                                         activation=tf.nn.sigmoid,␣
↪scope='deconv_2')(fold)

        # loss function
        loss = tf.nn.l2_loss(x - reconstruction)

        # training
        training = tf.train.AdamOptimizer(1e-4).minimize(loss)

        self.x = x
        self.reconstruction = reconstruction
        self.encoded = encoded
        self.loss = loss
        self.training = training
        self.name = "CAE-fs{}-fn{}-es{}".format(filter_size, filter_num,␣
↪encoded_size)


    def train(self, data, batch_size, passes, need_print = True):
        with tf.Session() as sess:
            saver = Model.start_new_session(sess)

            # start training
```

```
            for step in range(1, 1 + passes):
                for batch_i in range(data.shape[0] // batch_size):
                    train = data[batch_i * batch_size : (batch_i + 1) *␣
↪batch_size, :, :]
                    self.training.run(feed_dict={self.x: train})

                if (need_print and step % 200 == 0) or step == passes:
                    loss = self.loss.eval(feed_dict={self.x: train})
                    print("pass {}, training loss {}".format(step, loss))

                if step == passes:  # save weights
                    saver.save(sess, 'saver/{}'.format(self.name))


    def reconstruct(self, data, pid, save = False):
        def weights_to_grid(weights, rows, cols):
            height, width, in_channel, out_channel = weights.shape
            padded = np.pad(weights, [(1, 1), (1, 1), (0, 0), (0, rows * cols -␣
↪out_channel)],
                            mode='constant', constant_values=0)
            transposed = padded.transpose((3, 1, 0, 2))
            reshaped = transposed.reshape((rows, -1))
            grid_rows = [row.reshape((-1, height + 2, in_channel)).
↪transpose((1, 0, 2)) for row in reshaped]
            grid = np.concatenate(grid_rows, axis=0)

            return grid.squeeze()

        with tf.Session() as sess:
            saver, model_name = Model.get_particular_session(sess, self.name)

            # visualize results
            batch_size = 8
            x = data[(batch_size * pid):(batch_size * (pid + 1)), :, :, :]
            org, recon = sess.run((self.x, self.reconstruction),␣
↪feed_dict={self.x: x})

            input_images = weights_to_grid(org.transpose((1, 2, 3, 0)), 2, 4)
            recon_images = weights_to_grid(recon.transpose((1, 2, 3, 0)), 2, 4)

            fig, (ax0, ax1) = plt.subplots(ncols=2, figsize=(50, 25))
            ax0.imshow(input_images, cmap=plt.cm.gray, interpolation='nearest')
            ax0.set_title('input images', fontsize=40)
            ax1.imshow(recon_images, cmap=plt.cm.gray, interpolation='nearest')
            ax1.set_title('reconstructed images', fontsize=40)

            ax0.set_yticklabels([])
```

```
            ax0.set_xticklabels([])
            ax1.set_yticklabels([])
            ax1.set_xticklabels([])

            if save:
                plt.savefig('results/{}_reconstructed.png'.format(self.name),␣
↪bbox_inches='tight')
            else:
                plt.show()

    def getLatentVariable(self, data):
        with tf.Session() as sess:
            saver, model_name = Model.get_particular_session(sess, self.name)
            org, encoded = sess.run((self.x, self.encoded), feed_dict={self.x:␣
↪data})

        return encoded
```

[39]:

```
learning_rate = 1e-4
decay_weight = 1
filter_size = 3
filter_num = 64
encoded_size = 15

batchsize = 8
epoch_pass = 20000
```

3 Sliding window with weight decaying

[40]:

```
Sliding_Decayed_X = []
for window in Sliding_X:
    row_count = 6
    new_window = []
    for k in range(7):
        new_window.append(window[k, :, :] * (decay_weight ** row_count))
        row_count -= 1
    Sliding_Decayed_X.append(new_window)
Sliding_Decayed_X = np.array(Sliding_Decayed_X)
```

4 Training

[41]:

```
print("Start Training {}-{}-{}".format(filter_size, filter_num, encoded_size,␣
↪learning_rate))
conv_autoencoder = RevisedConvolutionalAutoencoder(filter_size, filter_num,␣
↪encoded_size, learning_rate)
```

```
conv_autoencoder.name = conv_autoencoder.name + "decay_weight_{}".
 ↪format(str(int(decay_weight * 100)))
conv_autoencoder.train(Sliding_Decayed_X, batch_size = batchsize, passes =␣
 ↪epoch_pass)
```

```
Start Training 3-64-15
started a new session
pass 200, training loss 36.63316345214844
pass 400, training loss 34.46383285522461
pass 600, training loss 33.32121276855469
pass 800, training loss 32.640098571777344
pass 1000, training loss 31.990737915039062
pass 1200, training loss 31.37820053100586
pass 1400, training loss 30.866107940673828
pass 1600, training loss 30.442338943481445
pass 1800, training loss 30.091012954711914
pass 2000, training loss 29.623825073242188
pass 2200, training loss 29.277557373046875
pass 2400, training loss 28.99906349182129
pass 2600, training loss 28.712425231933594
pass 2800, training loss 28.470041275024414
pass 3000, training loss 28.235187530517578
pass 3200, training loss 28.005273818969727
pass 3400, training loss 27.775665283203125
pass 3600, training loss 27.546070098876953
pass 3800, training loss 27.33101463317871
pass 4000, training loss 27.13214874267578
pass 4200, training loss 26.930204391479492
pass 4400, training loss 26.780017852783203
pass 4600, training loss 26.620994567871094
pass 4800, training loss 26.463050842285156
pass 5000, training loss 26.406299591064453
pass 5200, training loss 26.278568267822266
pass 5400, training loss 26.23134994506836
pass 5600, training loss 26.163280487060547
pass 5800, training loss 26.079805374145508
pass 6000, training loss 26.009851455688477
pass 6200, training loss 25.894296646118164
pass 6400, training loss 25.820842742919922
pass 6600, training loss 25.748456954956055
pass 6800, training loss 25.657390594482422
pass 7000, training loss 25.617389678955078
pass 7200, training loss 25.558155059814453
pass 7400, training loss 25.503145217895508
pass 7600, training loss 25.504764556884766
pass 7800, training loss 25.43017578125
pass 8000, training loss 25.335159301757812
pass 8200, training loss 25.32635498046875
```

```
pass 8400, training loss 25.295360565185547
pass 8600, training loss 25.302108764648438
pass 8800, training loss 25.253477096557617
pass 9000, training loss 25.182538986206055
pass 9200, training loss 25.09595489501953
pass 9400, training loss 25.079086303710938
pass 9600, training loss 25.03704833984375
pass 9800, training loss 25.05268096923828
pass 10000, training loss 25.018447875976562
pass 10200, training loss 24.969297409057617
pass 10400, training loss 24.90972137451172
pass 10600, training loss 24.885208129882812
pass 10800, training loss 24.897274017333984
pass 11000, training loss 24.897289276123047
pass 11200, training loss 24.82830810546875
pass 11400, training loss 24.757598876953125
pass 11600, training loss 24.8084659576416
pass 11800, training loss 24.683090209960938
pass 12000, training loss 24.68756103515625
pass 12200, training loss 24.59966468811035
pass 12400, training loss 24.52566146850586
pass 12600, training loss 24.457901000976562
pass 12800, training loss 24.497583389282227
pass 13000, training loss 24.449953079223633
pass 13200, training loss 24.421817779541016
pass 13400, training loss 24.43540382385254
pass 13600, training loss 24.413589477539062
pass 13800, training loss 24.372800827026367
pass 14000, training loss 24.412349700927734
pass 14200, training loss 24.419322967529297
pass 14400, training loss 24.396137237548828
pass 14600, training loss 24.41114044189453
pass 14800, training loss 24.42554473876953
pass 15000, training loss 24.41295051574707
pass 15200, training loss 24.442398071289062
pass 15400, training loss 24.518253326416016
pass 15600, training loss 24.376708984375
pass 15800, training loss 24.42007827758789
pass 16000, training loss 24.38104248046875
pass 16200, training loss 24.332672119140625
pass 16400, training loss 24.362274169921875
pass 16600, training loss 24.348588943481445
pass 16800, training loss 24.256389617919922
pass 17000, training loss 24.236316680908203
pass 17200, training loss 24.205110549926758
pass 17400, training loss 24.189117431640625
pass 17600, training loss 24.13921356201172
pass 17800, training loss 24.14886474609375
```

```
pass 18000, training loss 24.10614776611328
pass 18200, training loss 24.08757781982422
pass 18400, training loss 24.21141815185547
pass 18600, training loss 24.014667510986328
pass 18800, training loss 24.049917221069336
pass 19000, training loss 24.02139663696289
pass 19200, training loss 23.931236267089844
pass 19400, training loss 23.939002990722656
pass 19600, training loss 23.985580444335938
pass 19800, training loss 23.89499855041504
pass 20000, training loss 23.959964752197266
```

5 Save

6 Visualization (example)

[43]:
```
for i in range(4, 10):
    print("user id = {}".format(str(i)))
    conv_autoencoder.reconstruct(Sliding_Decayed_X, i, save = False)
```

```
user id = 4
INFO:tensorflow:Restoring parameters from saver/CAE-
fs3-fn64-es15decay_weight_100
restored model: CAE-fs3-fn64-es15decay_weight_100
```

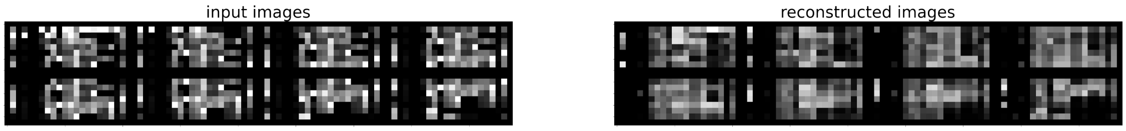


```
user id = 5
INFO:tensorflow:Restoring parameters from saver/CAE-
fs3-fn64-es15decay_weight_100
restored model: CAE-fs3-fn64-es15decay_weight_100
```

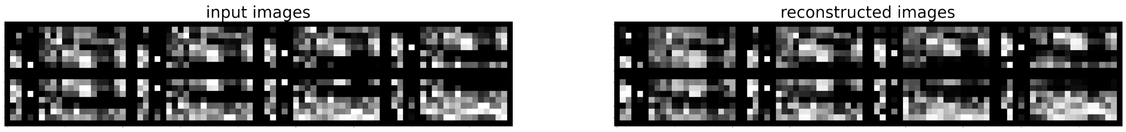


```
user id = 6
INFO:tensorflow:Restoring parameters from saver/CAE-
fs3-fn64-es15decay_weight_100
restored model: CAE-fs3-fn64-es15decay_weight_100
```

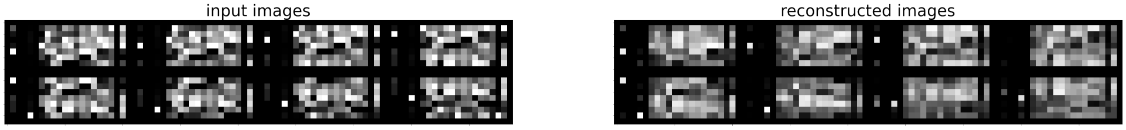


```
user id = 7
INFO:tensorflow:Restoring parameters from saver/CAE-
fs3-fn64-es15decay_weight_100
restored model: CAE-fs3-fn64-es15decay_weight_100
```

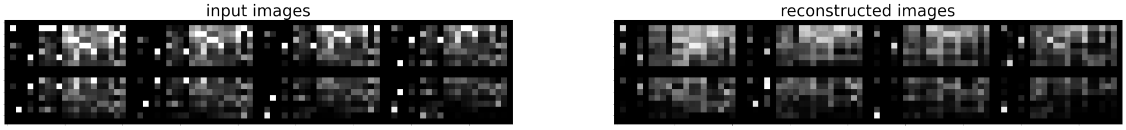


```
user id = 8
INFO:tensorflow:Restoring parameters from saver/CAE-
fs3-fn64-es15decay_weight_100
restored model: CAE-fs3-fn64-es15decay_weight_100
```

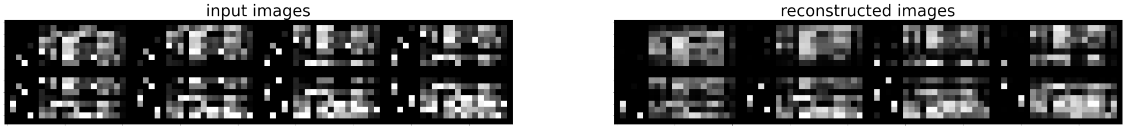


```
user id = 9
INFO:tensorflow:Restoring parameters from saver/CAE-
fs3-fn64-es15decay_weight_100
restored model: CAE-fs3-fn64-es15decay_weight_100
```

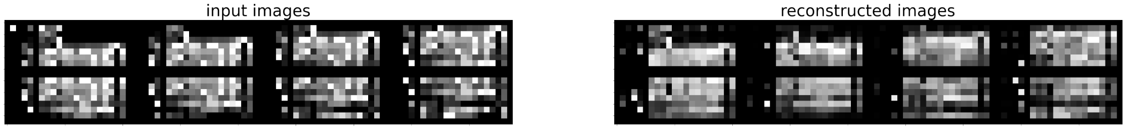

[ ]:
